# Enabling high-performance cloud computing for the Community Multiscale Air Quality Model (CMAQ) version 5.3.3: performance evaluation and benefits for the user community

**DOI:** 10.5194/gmd-17-7001-2024

**Published:** 2024-09-19

**Authors:** Christos I. Efstathiou, Elizabeth Adams, Carlie J. Coats, Robert Zelt, Mark Reed, John McGee, Kristen M. Foley, Fahim I. Sidi, David C. Wong, Steven Fine, Saravanan Arunachalam

**Affiliations:** 1 Institute for the Environment, The University of North Carolina at Chapel Hill, Chapel Hill, NC 27599, USA; 2 Research Computing, Information Technology Services, The University of North Carolina at Chapel Hill, Chapel Hill, NC 27599, USA; 3 Center for Environmental Measurement and Modeling, Office of Research and Development, U.S. Environmental Protection Agency, Research Triangle Park, NC 27709, USA

## Abstract

The Community Multiscale Air Quality Model (CMAQ) is a local- to hemispheric-scale numerical air quality modeling system developed by the U.S. Environmental Protection Agency (USEPA) and supported by the Community Modeling and Analysis System (CMAS) center. CMAQ is used for regulatory purposes by the USEPA program offices and state and local air agencies and is also widely used by the broader global research community to simulate and understand complex air quality processes and for computational environmental fate and transport and climate and health impact studies. Leveraging state-of-the-science cloud computing resources for high-performance computing (HPC) applications, CMAQ is now available as a fully tested, publicly available technology stack (HPC cluster and software stack) for two major cloud service providers (CSPs). Specifically, CMAQ configurations and supporting materials have been developed for use on their HPC clusters, including extensive online documentation, tutorials and guidelines to scale and optimize air quality simulations using their services. These resources allow modelers to rapidly bring together CMAQ, cloud-hosted datasets, and visualization and evaluation tools on ephemeral clusters that can be deployed quickly and reliably worldwide. Described here are considerations in CMAQ version 5.3.3 cloud use and the supported resources for each CSP, presented through a benchmark application suite that was developed as an example of a typical simulation for testing and verifying components of the modeling system. The outcomes of this effort are to provide findings from performing CMAQ simulations on the cloud using popular vendor-provided resources, to enable the user community to adapt this for their own needs, and to identify specific areas of potential optimization with respect to storage and compute architectures.

## 1 Introduction

Over the past decade, cloud computing has received a tremendous amount of attention for its potential to enable and simplify high-performance computing (HPC) applications. Modeling user communities can greatly benefit by having real-time access to cloud-ready reproducible workflows that include complex models and large datasets. Benefits can include the reduced effort required to manage computational resources, the ability to rapidly obtain more resources when needed, flexible approaches for managing costs and new opportunities for convenient data sharing. State-of-the-science numerical models simulating a variety of different processes and scales ranging from global circulation models to regional and high-resolution weather prediction workloads have been demonstrated to perform efficiently on HPC infrastructure in the cloud. Development groups for earth system models such as weather, climate, ocean circulation and air quality are currently designing and deploying modeling platforms or components that utilize different cloud environments (Campbell et al., 2023; Powers et al., 2021; Zhuang et al., 2020; Chui et al., 2019; Eastham et al., 2018; Chen et al., 2017).

The vast majority of such applications leverage “infrastructure as a code” (IaaC) or “infrastructure as a service” (IaaS) technologies and storage options provided by different cloud service providers, which creates the need for a flexible approach in terms of data integration. In the context of air quality models, cloud computing encapsulates both the data storage and parallel computing requirements for large-scale and high-resolution air quality simulations that frequently rely on output generated by other models that are dependent on chosen science configurations. Specifically, numerical models for simulating regional- and global-scale air quality events are developed with a core function to support a variety of science configuration options that are enabled at compile time in addition to a suite of run-time options. Efforts needed to treat such complex system models in the software as a service (SaaS) paradigm (Zhang et al., 2019) have remained exploratory and not gained enough traction, as correctly applying such models to specific situations demands a level of user control that goes beyond what is considered “power user” and involves administrative skills and in-depth HPC knowledge. This makes model deployment extremely difficult to achieve through a web-based interface. While end users have the option to use images with precompiled standardized versions of air quality models through cloud service provider (CSP) marketplace offerings at an hourly cost, these commercial products are designed for specific implementations and their associated base science options.

As an example, Zhuang et al. (2020) demonstrated the scalability of GEOS-Chem to thousands of cores using the Amazon^®^ Web Services (AWS) ParallelCluster to achieve similar computational and cost efficiencies of local HPC clusters. They provided an easy-to-follow research workflow in an HPC cluster environment on the cloud. We extended this work by running the Community Multiscale Air Quality Model (CMAQ) on AWS ParallelCluster and Microsoft Azure^®^ CycleCloud and using the HPC cluster highlevel frameworks or IaaC provided by these two major cloud providers. We provide tutorials that give end users the ability to reproducibly provision HPC clusters and software in a way that is optimized to run CMAQ on the cloud in a turn-key service.

Furthermore, the increase in availability of large datasets in the cloud through vehicles such as NOAA’s Big Data Program and NOAA Open Data Dissemination (Simonson et al., 2022; NOAA’s Big Data Program, 2023), Community Modeling and Analysis System (CMAS)’s Data Warehouse on AWS (CMAS’s Data Warehouse on AWS, 2023), and GEOS-Chem registry of open data (GEOS-Chem registry of open data, 2023) is another incentive to develop cloud solutions for air quality models that provide more leverage to the end user. Such initiatives are critical for the mission and growth of cloud modeling, and CSPs have acknowledged and addressed the emerging need of data democratization by waiving fees or providing free credits to facilitate access by scientists and average non-technical users of information systems. Tools such as AWS ParallelCluster and Azure CycleCloud are services that extend the power of IaaS by mimicking on-site HPC setups and provide an even more dynamically scalable environment that enables CMAQ modelers to step beyond the limits of single virtual machines (VMs), using the Simple Linux Utility Resource Management (Slurm) (Yoo et al., 2003) batch scheduler in a way that enables auto-scaling of the compute nodes, simplifying the cluster deployment and management. It is important to emphasize that ParallelCluster and Azure CycleCloud extend the capability from simply being able to run on a VM hosted in the cloud to a turn-key batch scheduling HPC tightly coupled cluster environment that is dedicated to the end user.

The Community Multiscale Air Quality Model (CMAQ) (Byun and Schere, 2006; Foley et al., 2010; Appel et al., 2017, 2021) is an open-source modeling system that consists of a family of programs for conducting air quality simulations and is being actively developed. The Community Modeling and Analysis System (CMAS) center facilitates community model development by hosting, developing and distributing software such as CMAQ; hosting the CMAS Center Forum (CMAS Center Forum, 2024) to facilitate the exchange of information related to code and datasets and troubleshooting; and providing outreach and support through new user training, annual conferences and workshops on specific topics. In many cases, one or more factors are increasing resource requirements for CMAQ simulations, including the addition of more complex algorithms to CMAQ, simulations of longer time periods or larger domains, and modeling grids with finer resolutions. For institutions that use traditional HPC centers, despite the evolution of job managers, resources frequently come with allocation time limits and long queue times. Even if groups can afford to acquire and maintain appropriate computing capacity, such an approach may not be cost-effective, especially if the capacity is not fully utilized. By leveraging cloud infrastructure, CMAQ users can pay monthly on-demand fees to perform model simulations on clusters managed by commercial providers without having to pay large up-front costs to purchase computer clusters or hire staff to maintain them. This can be extremely useful in enhancing computationally demanding research and air quality forecasting at an international scale, in many cases offering unprecedented expansion of such capabilities for developing nations. Another advantage of this approach is timely access to cutting-edge processors that otherwise would require disproportionate wait time, resources and effort to obtain. Similarly, scalability can be expanded in real time and with minimal effort.

The purpose of this study is to demonstrate the efforts required to bring CMAQ version 5.3.3 (US EPA Office of Research and Development, 2021) to the cloud and perform air quality simulations efficiently and affordably, leveraging existing and publicly available datasets. In the following sections, we describe several key aspects of this work:

- develop benchmark test suites that can address and replicate the needs of a typical CMAQ user
- streamline the CMAQ installation process in Amazon’s AWS and Microsoft Azure
- demonstrate running CMAQ on the cloud and estimate associated costs, making suggestions on different options available to the modeling community
- perform benchmark tests with different HPC clusters and their underlying VMs and networking and storage options while keeping track of the performance and associated costs
- make recommendations that would help reduce CMAQ simulation times specific to the cloud platform
- provide instructions for obtaining and using input datasets from the CMAS Data Warehouse under the AWS Open Data Sponsorship Program which waives data egress costs.

The methodologies used in this study are available as handson tutorials, with details for a variety of HPC systems on different CSPs, guides and recommendations for specific user needs (see the links under “HPC cluster deployment options”).

## 2 CMAQ workflow and cloud benchmark suite

Air quality modeling systems such as CMAQ rely heavily on the parameterization and simulation output from numerical weather prediction (NWP) systems in an offline coupling manner facilitated by preprocessing tools. Initial and boundary conditions for regional- to urban-scale simulations can be defined by the user to be either static or the result of nested downscaling from a coarser domain model application (i.e., Hemispheric CMAQ). A common CMAQ workflow involves

1. developing meteorological fields with the Weather Research and Forecasting (WRF) model (Skamarock et al., 2021)
2. processing WRF output using the Meteorology–Chemistry Interface Processor (MCIP) (Otte and Pleim, 2010)
3. developing emissions inputs using the Sparse Matrix Operator KErnel (SMOKE) modeling system (Houyoux et al., 2000)
4. developing other inputs such as initial and boundary conditions using preprocessors
5. performing air quality simulations using the complete set of inputs
6. assessing the successful completion of the simulation and verifying the model output and
7. analysis of the results to address the purpose of the simulation (e.g., regulatory or research issues).

Cloud storage enables reproducible workflows by having both model and datasets publicly available and directly accessed by the run scripts.

Traditionally, every CMAQ release is distributed with a lightweight test case that includes all inputs necessary for the user to confirm a successful installation and completion of a multi-day simulation. Similarly, a newly standardized test case, referred to as the cloud benchmark suite (CBS), was developed to evaluate CMAQ’s performance on cloud HPC environments. Benchmark suite simulations were designed considering different user needs and data availability to construct a well-established bundle of inputs and outputs that can be further scaled and customized to meet specific scalable requirements.

The hardware configuration necessary to run CMAQ depends on the domain size, grid resolution, complexity of physics and chemistry parameterization, number of variables and layers saved to the concentration and diagnostic files, and simulation duration. Since typical input and output datasets for CMAQ include 3D descriptions of the dynamical and chemical state of the simulated atmosphere, these datasets could require several gigabytes of disk storage per simulation day.

**Figure 1.**
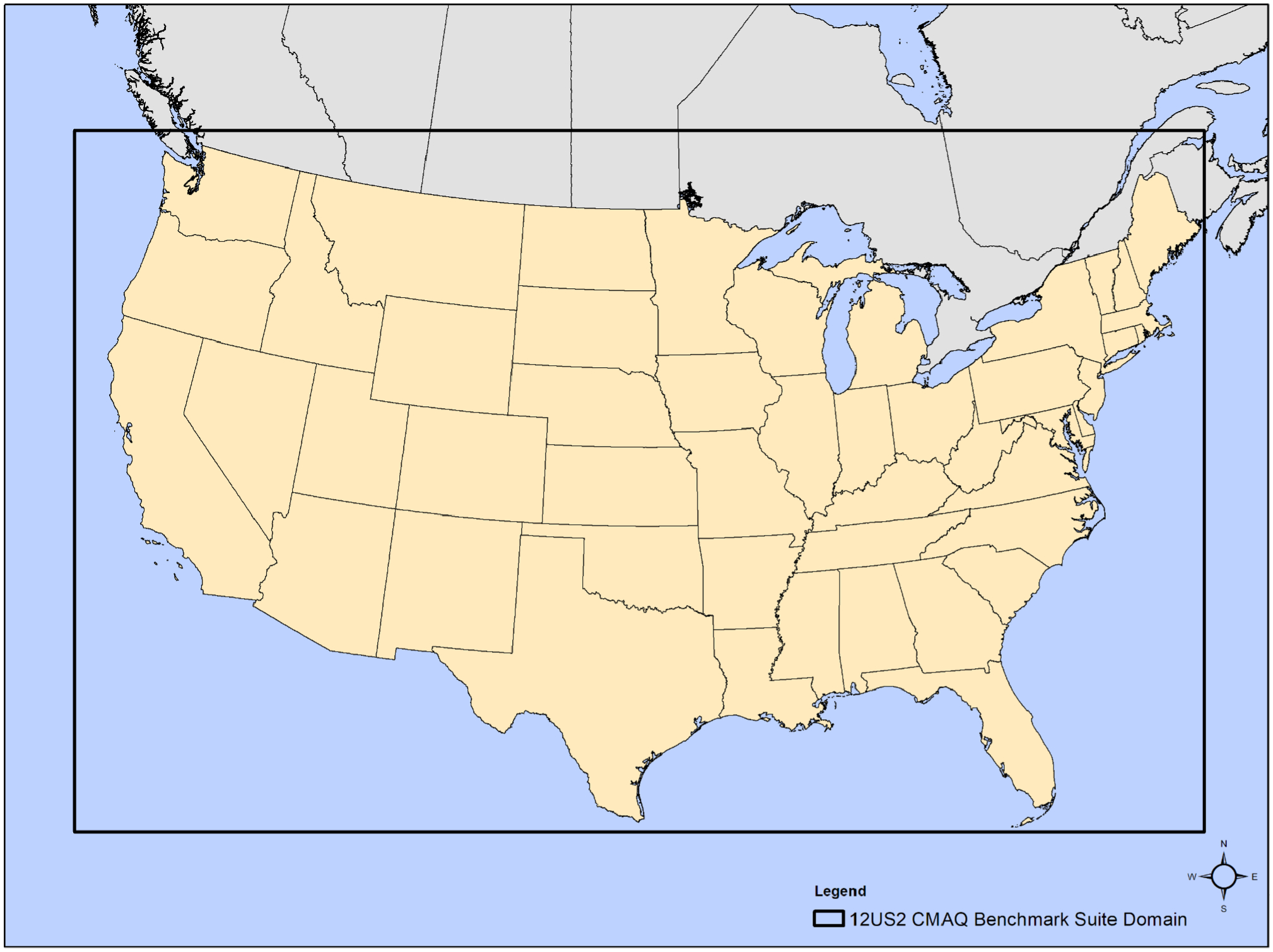
Cloud benchmark suite modeling domain (“12US2”; 396 columns by 246 rows by 35 vertical layers) for the CONUS at a 12 km × 12 km horizontal grid spacing is shown as the bold rectangle.

Given these considerations, a 2 d CBS for the contiguous United States (CONUS) was constructed with the aim to be representative of a commonly used domain over a time frame that can be used to fully test the CMAQ system. Typical requirements for a CONUS 12 km × 12 km horizontal grid resolution are provided in Table 1 below, while Fig. 1 shows a map created with ArcGIS with the domain’s coverage. (ArcGIS Pro, 2024).

**Figure 2.**
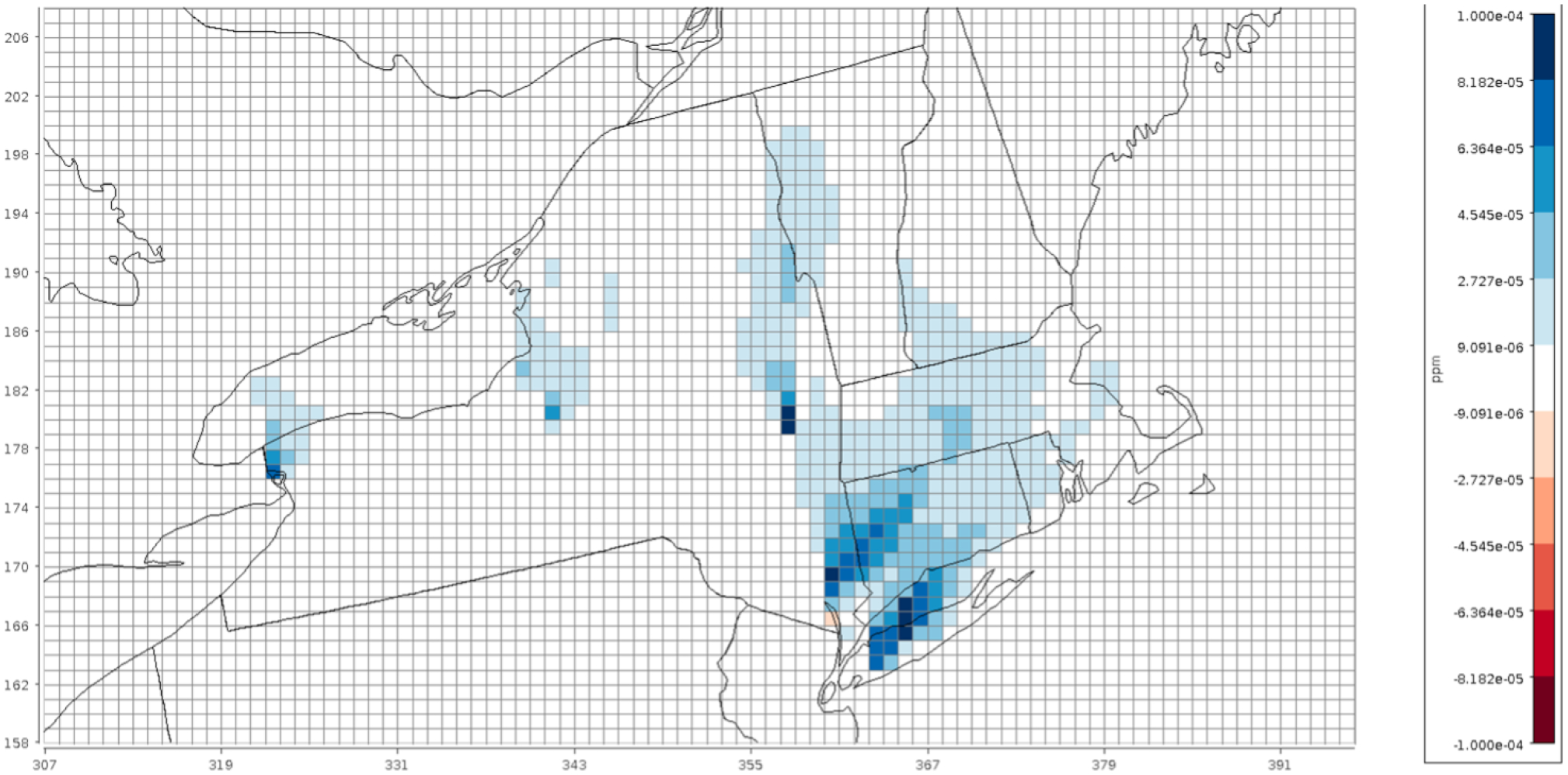
Spatial plot of differences in NO_2_ concentrations when power plant emissions in New York were reduced by 25 %. Lines represent the 12 km × 12 km model grid, while the *x* and *y* axes represent the row and column numbers of the model grid cells for the CONUS domain shown in Fig. 1.

All simulations used a modified version of CMAQ version 5.3.3 with the CB6 chemical mechanism and aerosol module 7 (cb6r3_ae7_aq). The Detailed Emissions Scaling, Isolation and Diagnostic (DESID) module was also used to reduce the emissions of a specific emission stream for a specific region within the domain to highlight this new emission-scaling capability offered within CMAQ (Murphy et al., 2021). Further details are provided later in this paper. Datasets are typically created in the NetCDF data format (Unidata, 2023), which allows for sharing on the cloud following programming methods that leverage the power of Models-3/EDSS Input/Output Applications Programming Interface (The BAMS/EDSS/Models-3 I/O API, 2023). Figure 2 shows a subset of the CONUS domain depicting the reduction in concentration of NO_2_ over the northeastern USA due to scaling of the point-source emissions from electric generating units (PT_EGU) in New York by 75 % (baseline minus sensitivity case emissions), achieved directly using the DESID module. This VERDI plot illustrates the resolution of the grid as compared to the state boundary lines (VERDI, 2024). The storage space requirements are defined based on the need to perform multiple sets of identical runs while changing the number of cores used to run CMAQ for single-node and parallel HPC clusters using OpenMPI (Gabriel et al., 2004) to evaluate scalability and to accommodate the additional disk space required for base and sensitivity runs.

Benchmark runs were performed with two output options: the first using a fully enabled concentration (CONC) output option (37 variables, 36 layers) and the second with a reduced number of variables and layers saved to the output concentration (CONC file) (12 variables, 1 layer). The scaling benchmarks used the reduced file option because the I/O API in its current version is not parallelized, and using the full-output file may have negatively impacted the compute portion of scaling.

**Figure 3.**
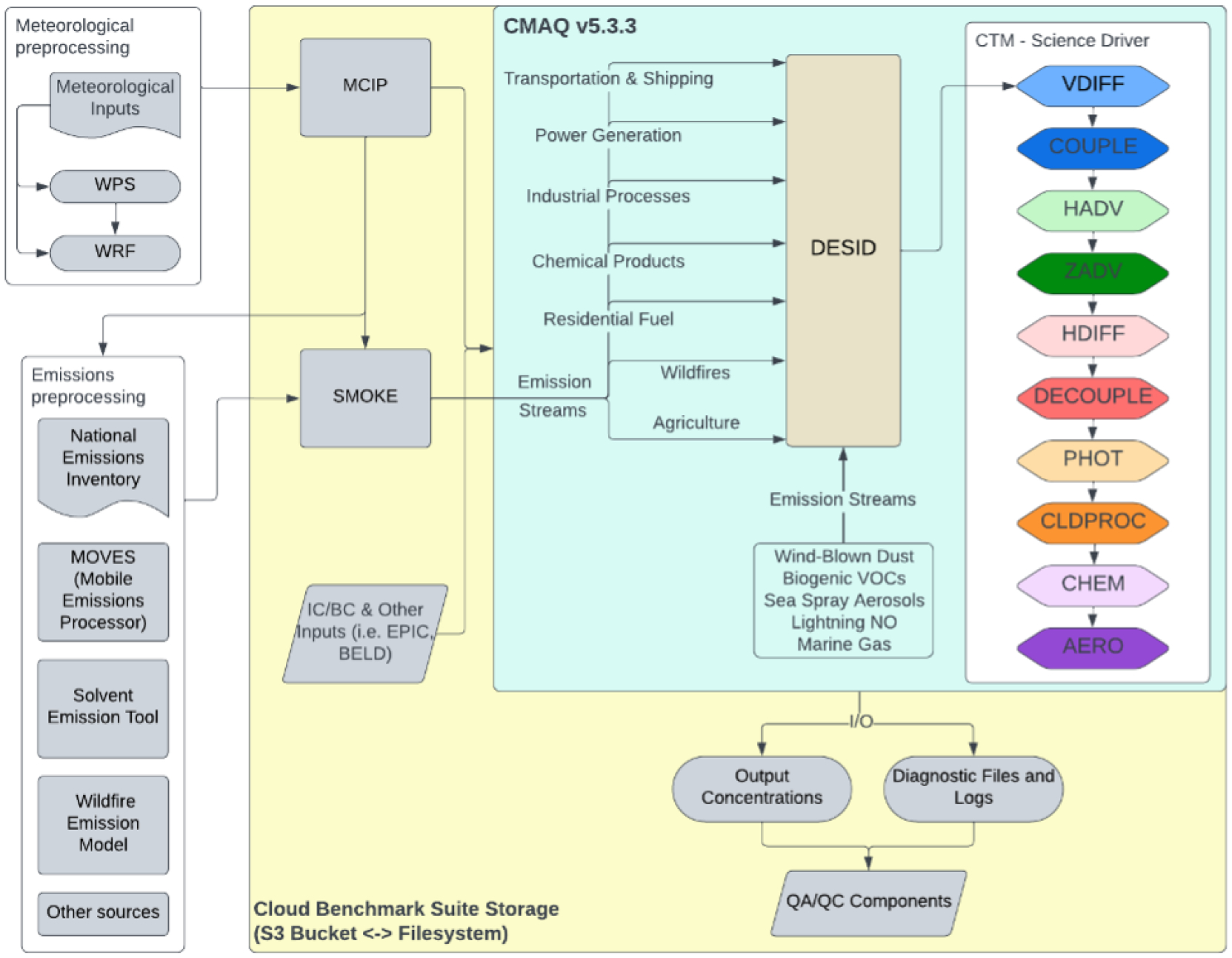
CMAQ flowchart (CTM science driver processes are color coded to match timings captured in Figs. 8–12).

Figure 3 shows the flowchart created with LucidChart of how to run CMAQ, with the chemical transport model (CTM) scientific processes color coded to match the benchmark timing equivalent found in the main log file in Figs. 8–12 (Lucidchart, 2024).

## 3 CMAQ experimental design for CSPs

### 3.1 CMAQ software stack

#### Model and prerequisite libraries.

**Figure 4.**
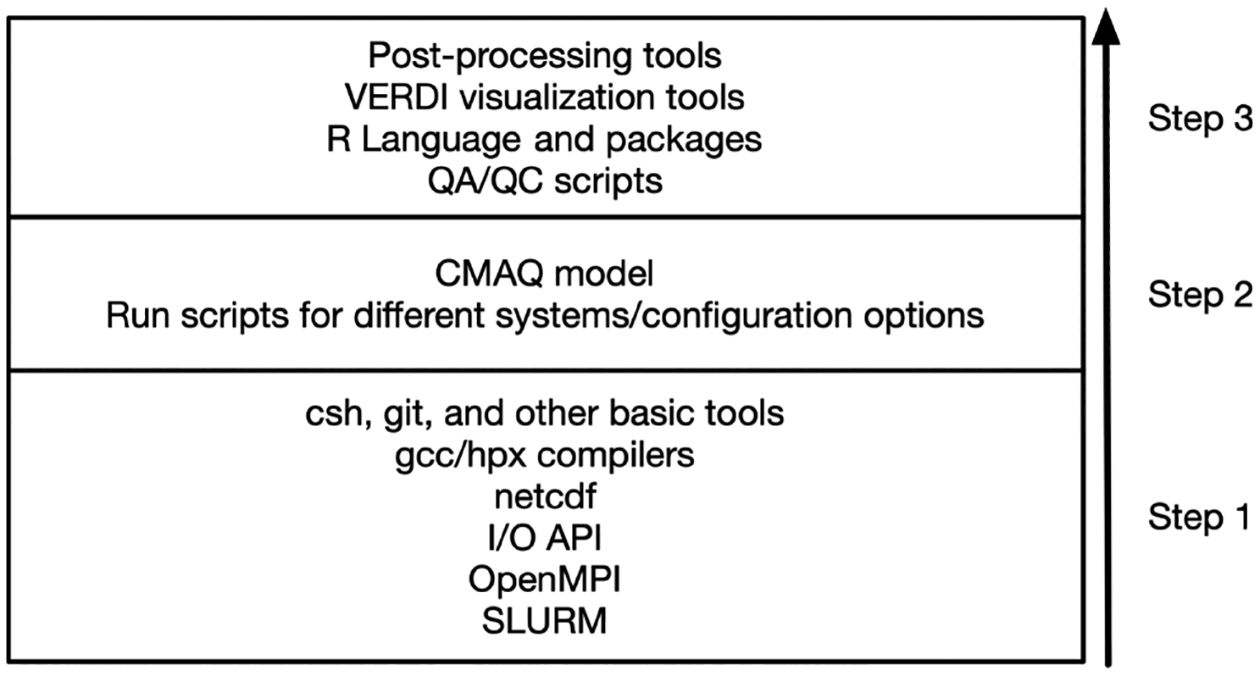
Multi-step approach in installing CMAQ prerequisites, model code, postprocessing, and other visualization and evaluation tools of the software stack.

Installing and setting up CMAQ on different CSPs with comparable Linux operating systems follow the general method depicted in the schematic of Fig. 4. Step-by-step instructions to install the software stack using automated C-shell scripts are provided in the online tutorials. In addition, the tutorial covers the preparation of the benchmark data and provides run scripts for launching CMAQ through the job manager. To facilitate an even better approach, publicly available snapshots of the “/shared” volume that contains the software stack are provided for each CMAQ version and hardware release. This allows new users to build clusters and quickly run CMAQ on HPC systems on the cloud. Additionally, it allows users to directly invoke existing precompiled libraries as modules, allowing for multiple applications and versions to be used and speeding up model workflows and modifications (https://modules.readthedocs.io/en/latest/, last access: 20 June 2024).

Depending on networking and storage options, users may need to add specific drivers and/or file system clients/layers to the list of installed modules. In parallel file system cases like Lustre, a client that is OS-specific needs to be present and linked to a storage account associated with the cluster to proceed for Azure CycleCloud. AWS offers a built-in Lustre implementation for most of their VMs including ParallelCluster. Azure VM images with embedded Lustre clients linked to a Lustre volume, currently in a public beta testing phase, were made available for our benchmark cases.

#### Data transfer options.

AWS VMs have the AWS Command Line Interface (CLI) that is used to copy data from the S3 buckets available to the public through the AWS Open Data Sponsorship Program. For the case of Azure, users are provided instructions to install and use the AWS CLI and a *csh* script to copy the data from the CMAS Data Warehouse on the AWS Open Data S3 bucket to the storage option being used. Data could also be copied from non-public S3 buckets to which the user has access privileges. An alternative is to link the S3 bucket to Lustre on AWS or create blob storage on Azure and connect that blob storage to Lustre directly to speed up access to input data. Azure users may want to use datasets from Microsoft’s AI for Earth Data Sets (https://microsoft.github.io/AIforEarthDataSets/, last access: 20 June 2024).

#### HPC cluster deployment options.

**Figure 5.**
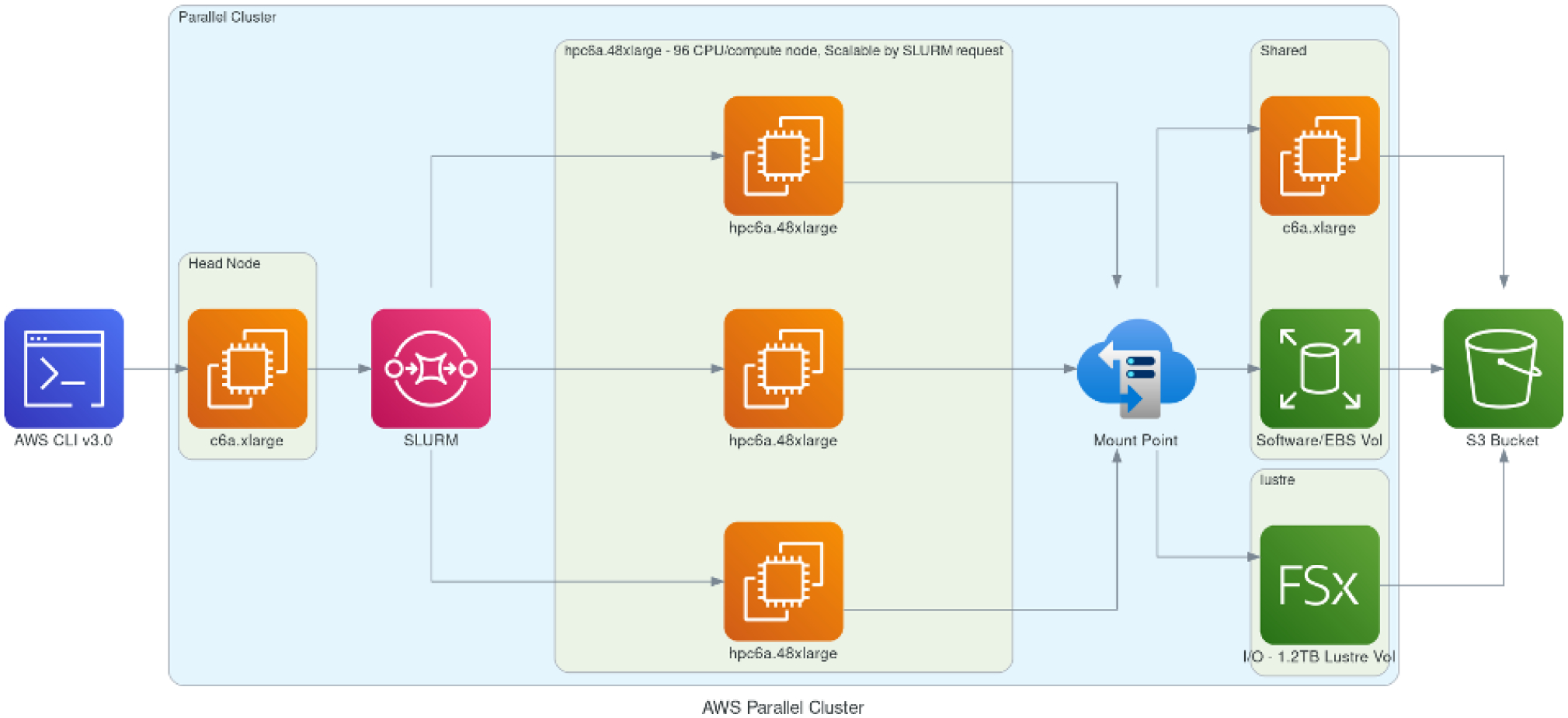
Schematic demonstrating AWS’s ParallelCluster-based framework utilizing elastic compute nodes scalable by SLURM request and different networked storage options (EBS, Lustre) with archival storage to AWS S3 bucket. Created with Open Source Diagrams (Kwon, 2024).

**Figure 6.**
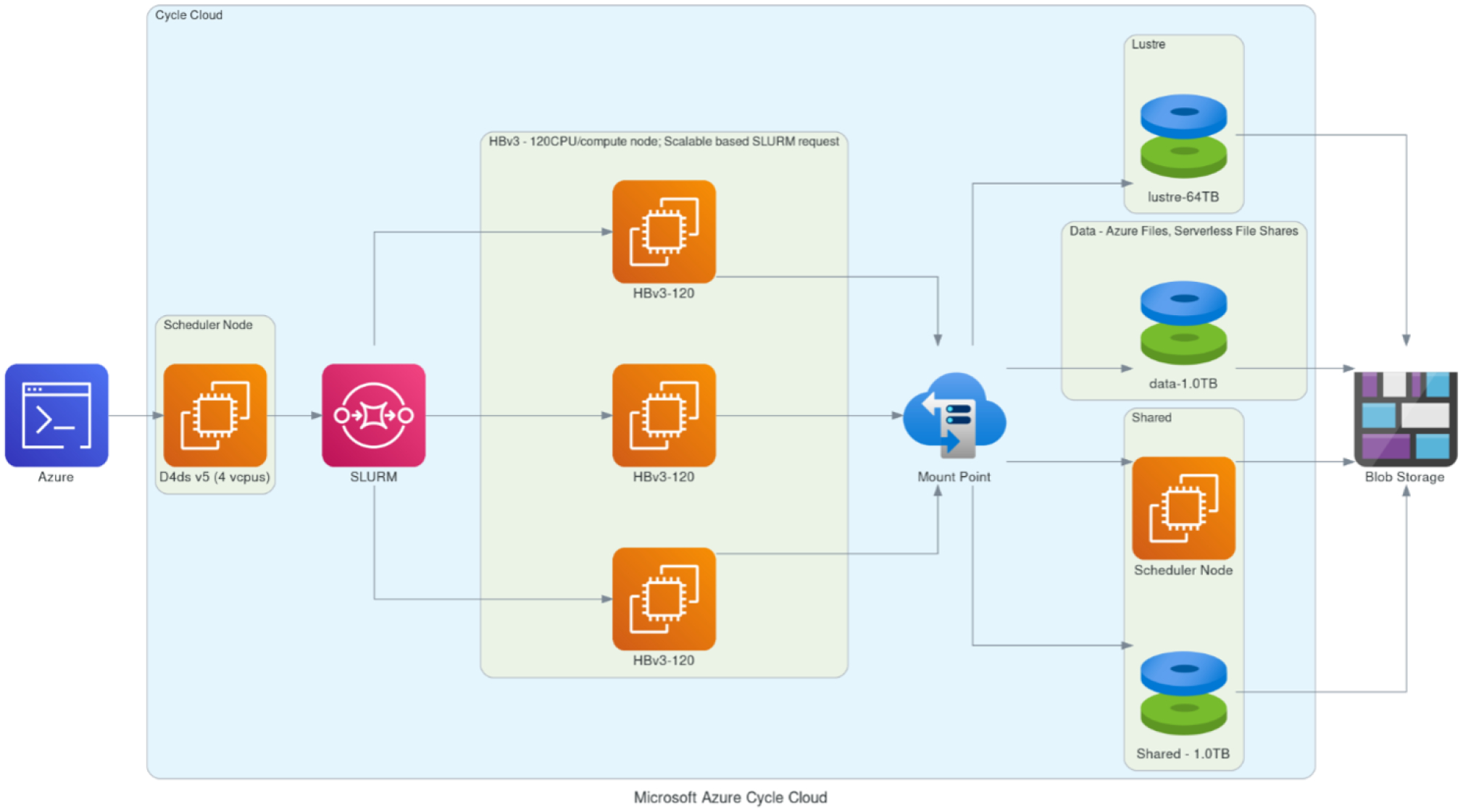
Schematic demonstrating Microsoft Azure’s CycleCloud-based framework utilizing compute nodes scalable by SLURM request with different networked storage options (Lustre, data, shared) with archival storage to Azure Blob Storage. Created with Open Source Diagrams (Kwon, 2024).

Step-by-step guidance for each CSP and the workflow used to run the benchmark has been documented and provided in the following tutorials (Azure: https://cyclecloud-cmaq.readthedocs.io/en/latest/, last access: 20 June 2024; AWS: https://pcluster-cmaq. readthedocs.io/en/latest/, last access: 20 June 2024). A verbose section was included in the run script structure to allow for recording architecture and OS-specific parameters in the log files, including higher-precision time tracking of each model process. Recommendations for optimal MPI process placement using the Slurm Workload Manager with pinning on Azure HB-series VMs were established for CycleCloud applications (https://techcommunity.microsoft.com/t5/azure-high-performance-computing/optimal-mpi-process-placement-for-azure-hb-series-vms/ba-p/2450663, last access: 20 June 2024). Process placement was also used for ParallelCluster applications on AWS, optimized for the Hpc6a series. In the process outlined in Fig. 4, we have also included code profiling tools (e.g., ARM^®^ MAP – https://www.linaroforge.com/, last access: 20 June 2024) which allow for a better understanding of code performance and optimization opportunities for various applications/problem sizes. In Figs. 5 and 6, we present overview schematics of the single VM and cluster configuration in each CSP. With respect to storage options, we chose the naming convention /lustre to refer to running CMAQ and saving the output on a Lustre parallel file system on AWS and Azure, “/shared” for using Elastic Block Store (EBS) on AWS and built-in network file system (NFS) volume with default configuration on Azure, and “/data” for Azure’s external NFS share option (for more information see https://learn.microsoft.com/en-us/azure/storage/common/nfs-comparison, last access: 20 June 2024, and https://docs.aws.amazon.com/parallelcluster/latest/ug/SharedStorage-v3.html, last access: 20 June 2024). In general, storage implementations are CSP-specific and have different performance characteristics and fine-tuning options.

#### HPC cluster monitoring options.

The AWS CloudWatch (https://aws.amazon.com/cloudwatch, last access: 20 June 2024) web page interface was used to monitor and compare the throughput of the I/O on the EBS and Lustre file systems using the full-output 37 variables and all layers in the CONC file. The Azure Monitor Metrics (https://learn.microsoft.com/en-us/azure/azure-monitor/essentials/data-platform-metrics, last access: 20 June 2024) web page interface was used to compare the latency and throughput of the I/O on the shared and Lustre file systems using the cloud benchmark suite (CBS_full).

### 3.2 CSP computation options

The first step to begin using the cloud is to engage a cloud service provider (CSP) and create an account. This is the user’s responsibility, and CSPs have direct dedicated support to address specific user needs. Cloud-based CMAQ setups were developed and are currently available on two CSPs: Amazon (AWS) and Microsoft (Azure). Typical CMAQ modeling workflows on the cloud can be divided into two general approaches: provisioning a single virtual machine and provisioning a dynamic multi-node cluster system. As multiprocessing architectures have evolved, many vendors are offering single VMs with more than 100 CPU cores, making them ideal for flexibly allocating and managing resources for computational simulations while limiting the effort required to compile and maintain the code and scripts. Clusters can be created in a multi-node framework following a similar approach once access and the availability of the total amount of resources are granted by the CSP.

After a thorough initial testing of the model code with a wide spectrum of hardware options offered by cloud vendors for HPC applications, we established the best-performing architecture configurations described in Table 2 as the hardware stack test bed for final benchmarking in this study. Amazon’s Hpc6a instances are powered by two 48-core third-generation AMD EPYC 7003 series processors built on 7 nm process nodes for increased efficiency, 384 GB of memory and 256 MB of L3 cache with a total of 96 cores. AWS Nitro System offloads the hypervisor to a dedicated card that also provides high-speed networking and high-speed Elastic Block Store (EBS) services, allowing all 96 cores of the AMD chip to be used for simulations (AWS, 2023). Azure’s HB120v3 server features two 64-core AMD EPYC 7V73X processors for a total of 128 physical cores, while each section contains 8 processor cores with uniform access to a 96 MB L3 cache/section. The Azure HB120v3 was designed to reserve 8 cores for the hypervisor and provides the remaining 120 cores for the application. Modern processors such as AMD’s EPYC series employ non-uniform memory access, a multiprocessing (multi-die) architecture in which each processor is attached to its own local memory (called a NUMA domain) but can also access memory attached to another processor. To maximize the performance for each AMD chip it is important to balance the amount of L3 cache and memory bandwidth per core at the job level. This means that the binding of a process or thread to a specific core, known as CPU pinning or processor affinity, will now have to include additional steps for NUMA topology optimization (Podzimek et al., 2015; Ghatrehsamani et al., 2020).

### 3.3 Networking options

In the tutorials and code implementations, we employed CSP-specific advanced networking options that reflect the available hardware options, enabling the 100 Gb s^−1^ Elastic Fabric Adapter (EFA) on AWS and the 200 Gb s^−1^ high-dynamic-range (HDR) InfiniBand on Azure to support the level of high-performance computing required by CMAQ.

### 3.4 Storage options

For storage, the real-time allocation of bandwidth and input/output operations per second (IOPS) differs between cloud vendors and should be examined independently at the application level by the user. In the examples investigated in this study, the user has access to four CSP-specific types of storage:

1. the fastest built-in local storage using nonvolatile memory express solid-state drives (NVMe SSDs) that are included with default single-VM provisioning
2. network file systems tied to the user/enterprise account accessible using the network file system (NFS) for Azure and elastic file system (EFS) for AWS, attached to the head node in a cluster environment or directly to a VM
3. unique services such as AWS’s Elastic Block Store (EBS) which are designed for per-instance blocks for certain compute cloud frameworks such as single AWS elastic cloud (EC2) and Azure’s NetApp Files (ANF)
4. fully managed high-performance file system such as Lustre developed for HPC cluster environments (also tested with single VMs) – Lustre implementations offering improved performance and allowing for multiple compute servers to connect to the Lustre host, where several servers are responsible for handling the transfer.

#### Cloud HPC configuration summary.

We explored all the above options to have a complete set of solutions for different model cases and user needs that can be formulated around the cloud benchmark suite. In the standard CMAQ implementation, input is read by all available cores, while output is handled by only one of them. While the model performed as expected with single VMs, the code base had to be modified to correct issues with NFS-mounted storage in cluster environments that utilize more than ~ 180 cores. The code changes did not have an impact on the model results. If a parallel file system is present (i.e., Lustre, BeeGFS), users have the option to configure CMAQ with the parallel I/O algorithm (Wong, 2024). Such implementations for CMAQ have been explored in previous versions of the model code base, and performance was investigated in more I/O-demanding, higher spatially resolved simulations (Wong et al., 2015) that need to be thoroughly tested on the cloud with current compilers and hardware and were not considered at this stage of model benchmarking. It is, however, important to note that CMAQ input and output file sizes are highly dependent on the domain size and output file configuration options that can be simulation-specific, and users are encouraged to perform further analysis for their unique modeling application needs. Table 3 summarizes the different storage options that were included in the final set of benchmarks. This list does not include certain storage solutions such as Azure NetApp Files (ANF), common internet file shares (CIFS) and the BeeGFS parallel file system, as these options either were deemed too expensive or created challenges when benchmarking CMAQ; e.g., CIFS does not allow for file links, ANF was more expensive for the CMAQ paradigm compared to other offerings from Microsoft, and BeeGFS is not available as a service and needs additional server setup and tuning. The cluster configurations are described in Figs. 5 and 6 as they demonstrate how the HPC resources on the cloud are dynamically provisioned by the ParallelCluster and CycleCloud user interface. Figure 5 shows that the user logs into a head node on AWS and submits a CMAQ run using the SLURM scheduler. The HPC6a compute nodes are deployed only when CMAQ is running, with the number of nodes deployed specified by the SLURM commands within the run script. The other details of the configuration of the compute cluster including the type of head node, type of compute node, and type of networking and storage available are specified in the YAML file that was used to create the cluster. As shown in Fig. 6, the user also logs into a head node on Microsoft Azure, with the number of compute nodes provisioned when the CMAQ job is running within the SLURM queue. The selection of head node, compute node, networking and storage type is made through a web interface to the Azure CycleCloud user interface (UI).

## 4 Results

### 4.1 Single-node VM timing analysis

The CMAQ simulation will write two types of log files: a main log file and processor-specific log files for each core/process. Model performance was evaluated using the main log files that include timings for the major science modules at each time step: vertical diffusion (VDIFF), COUPLE (converts units and couples or decouples concentration values with the density and Jacobian for transport), horizontal advection (HADV), vertical advection (ZADV), horizontal diffusion (HDIFF), DECOUPLE, photolysis (PHOT), cloud process (CLDPROC), chemistry (CHEM) and aerosol (AERO). Horizontal advection is the most time consuming of the processes within CMAQ. This is most likely due to communication between processors during advection, which requires information from neighboring cells to calculate advective fluxes. This is domain-dependent, and there can be domains where the computational demand is very large (e.g., applications like Model Prediction Across Scales (MPAS); Gilliam et al., 2021) so that one may not see this trend till one uses thousands of cores. In short, more cores result in less work per core, but more time is needed for each core to communicate with each other. At the end of each simulation hour, species concentrations are output along with the timings printed for the output process (data output). It should be noted that this output process timing does not fully capture the total I/O time including initializing and shutting down the model (i.e., close all files, deallocate arrays). This unaccounted time component is derived from the difference between the total wall time (elapsed real time) and the sum of the sub-processes and was labeled as OTHER in the parse_timing R plots (Bash, 2024).

**Figure 7.**
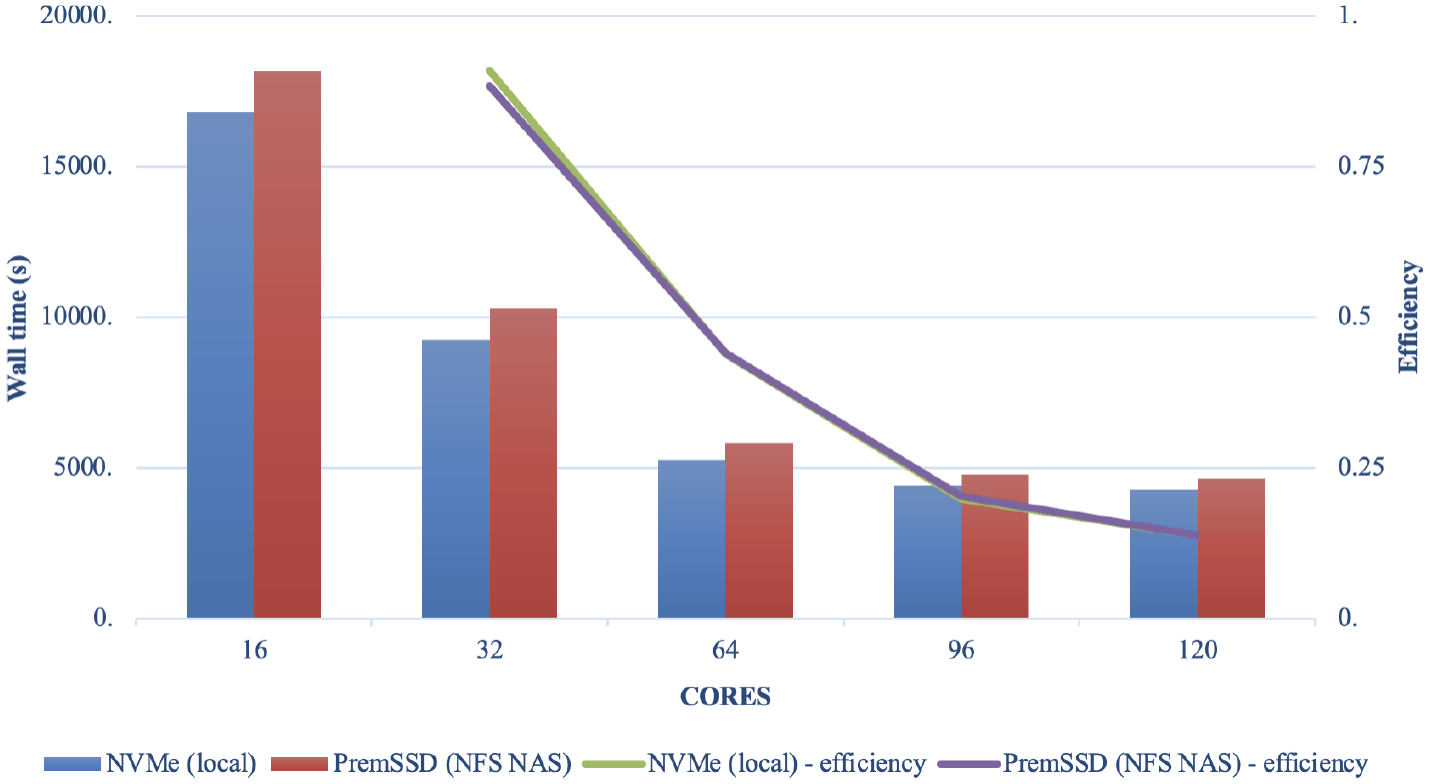
Performance comparison of the cloud benchmark suite (CBS_limited) simulations on a single VM of Microsoft Azure utilizing 16–120 cores with a fast local SSD NVMe and premium SSD through the NFS client.

**Figure 8.**
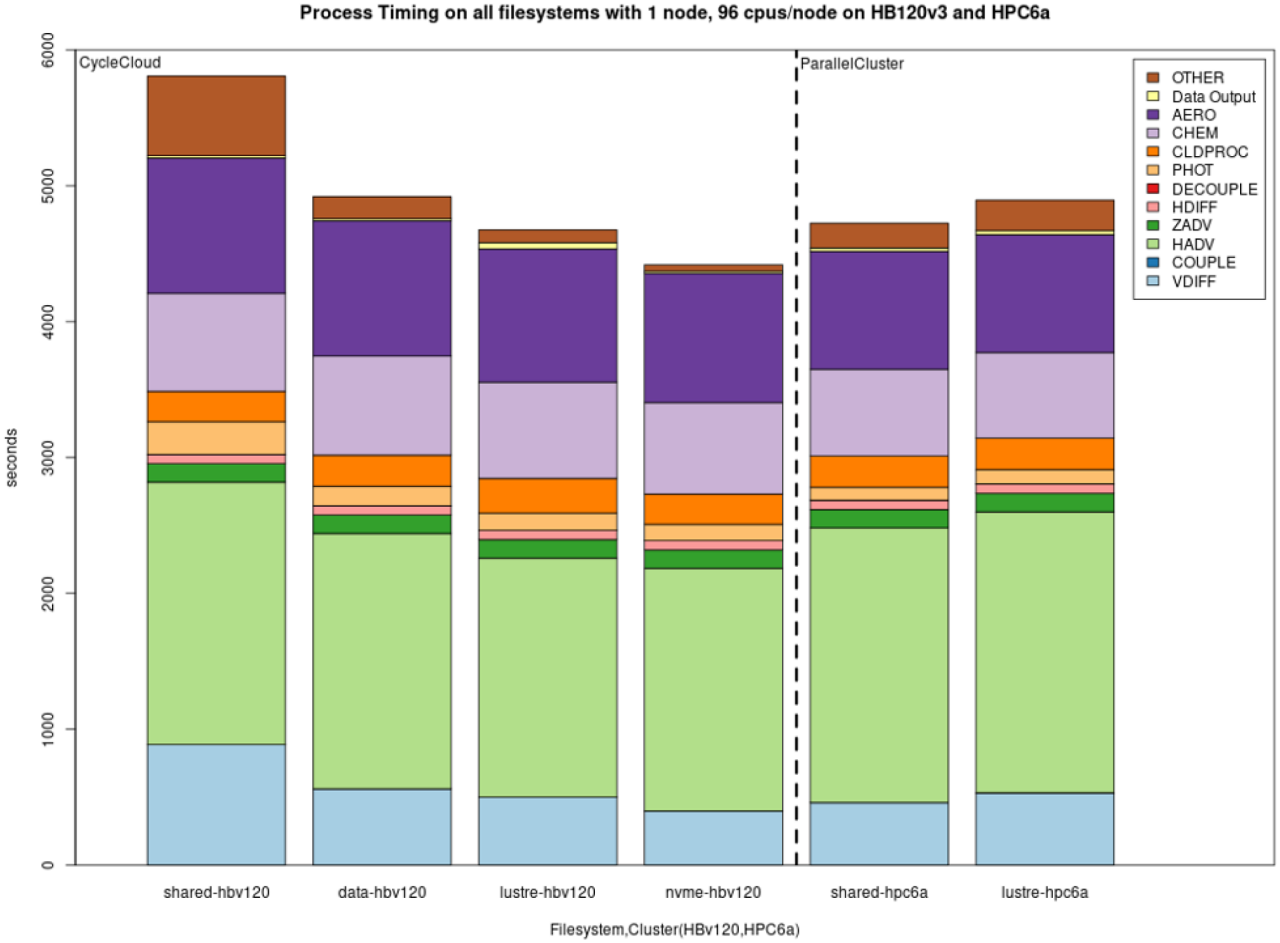
Performance comparison per model component for the cloud benchmark suite (CBS_limited) on a single node of Microsoft Azure utilizing 96 cores with a premium SSD through the NFS client (shared), a NetApp Files (ANF) solution (data), a Lustre file system and a fast local SSD NVMe, as well as on a single node of AWS ParallelCluster utilizing 96 cores with the EBS (shared) and Lustre file system solutions (from left to right).

**Figure 9.**
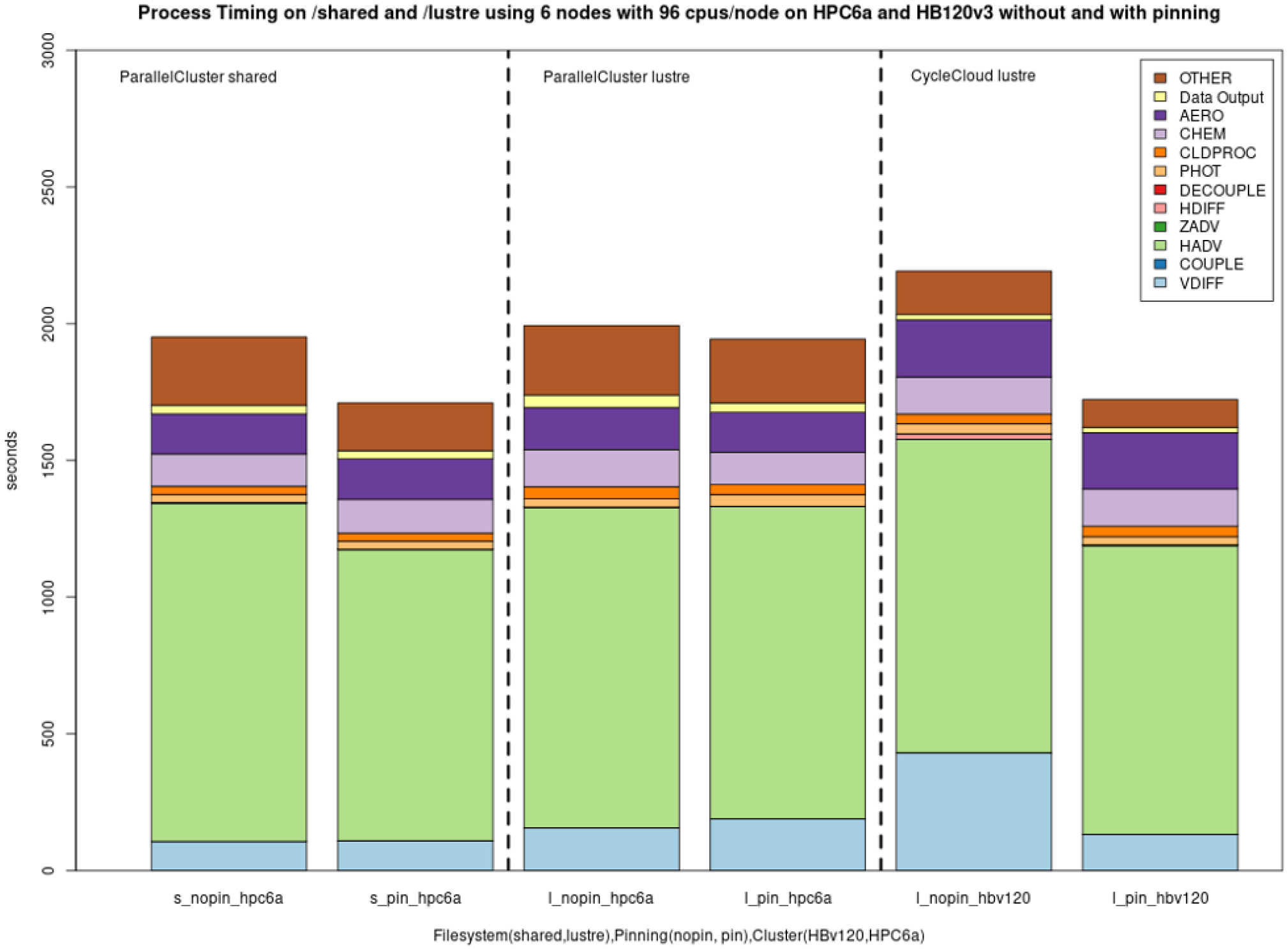
Effect of process pinning on /shared and /lustre and on AWS ParallelCluster (HPC6a) (576 cores) and on Azure CycleCloud HBv120 (576 cores) on /lustre for cloud benchmark suite (CBS_limited).

Model scalability is the measure of a system’s ability to increase (or decrease) performance (and cost) in response to changes in system processing power, in our case determined by the specific resources (cores, memory, storage, and network protocols and bandwidth), and relies on MPI implementation and integration with the job manager (Slurm). Results from the benchmark case simulations performed in a single-node EPYC VM of Microsoft Azure are presented in Fig. 7. Figure 7 demonstrates good performance and efficiency scaling with both local and NFS solid-state drive (SSD) storage options and some degree of a leveling off observed above 96 cores. As expected, the fastest local NVMe solution performs better than the same system with different storage options. Since NVMe is included in the default configuration, it is also the cheapest solution for a testing phase, and despite its fixed volume it is sufficient for the benchmark domain and simple user needs (i.e., benchmarking, code development, testing). For larger domains and simulation periods, the SSD over NFS is a preferred solution that allows for larger volumes of data to be attached. Figure 8 provides a cloud benchmark case performance comparison broken down by model process component for each storage solution within Azure and AWS. A difference in the VM core allocation for hypervisor and background system tasks results in a different core count available for computing between the CSPs. For direct comparison with AWS, the system in both CSPs was optimized to utilize 96 out of the 120 available cores by employing process pinning, matching the region of best scalability observed in Fig. 7. From Fig. 8, it is evident that on Azure the Lustre file system competes very well with the local SSD solutions, followed by an additional performance difference for the NFS share (/data) and the proportionally slower but cheaper built-in NFS storage. On AWS, we could not provision a local SSD for the hpc6a.48xlarge single VM, so benchmark tests were limited to Lustre and EBS storage options. Results depicted in Fig. 8 indicate that Lustre on AWS performed slightly slower compared to Azure, while the EBS option was a faster and more cost-effective solution for this CSP. However, users are advised to copy the input and output data in EBS (and local solution on Azure) as part of their workflow, and additional time to complete the simulation should be accounted for.

### 4.2 Optimization and benchmarking on multi-node clusters

#### 4.2.1 Process pinning for L3 cache optimization in the EPYC processor architectures

As mentioned before, in a managed job environment, AMD EPYC processors offer an option called “process pinning” that can improve performance through more effective use of the L3 memory cache at the job submission level. This is another configuration option that should be evaluated, especially since implementations vary between CSPs and may change over time. Figure 8 demonstrates the effect of process pinning on AWS and Azure. This option reduced simulation times on both systems using Lustre and shows that this effect can vary depending on the file system as well, with EBS volume use pointing to more substantial performance gains for AWS. Nevertheless, both Azure and AWS users should carefully consider such performance gains and further evaluate scaling under different pinning options for their domain and configuration.

#### 4.2.2 Results on multi-node clusters with different storage options saving 12 variables to the one-layer CONC file

**Figure 10.**
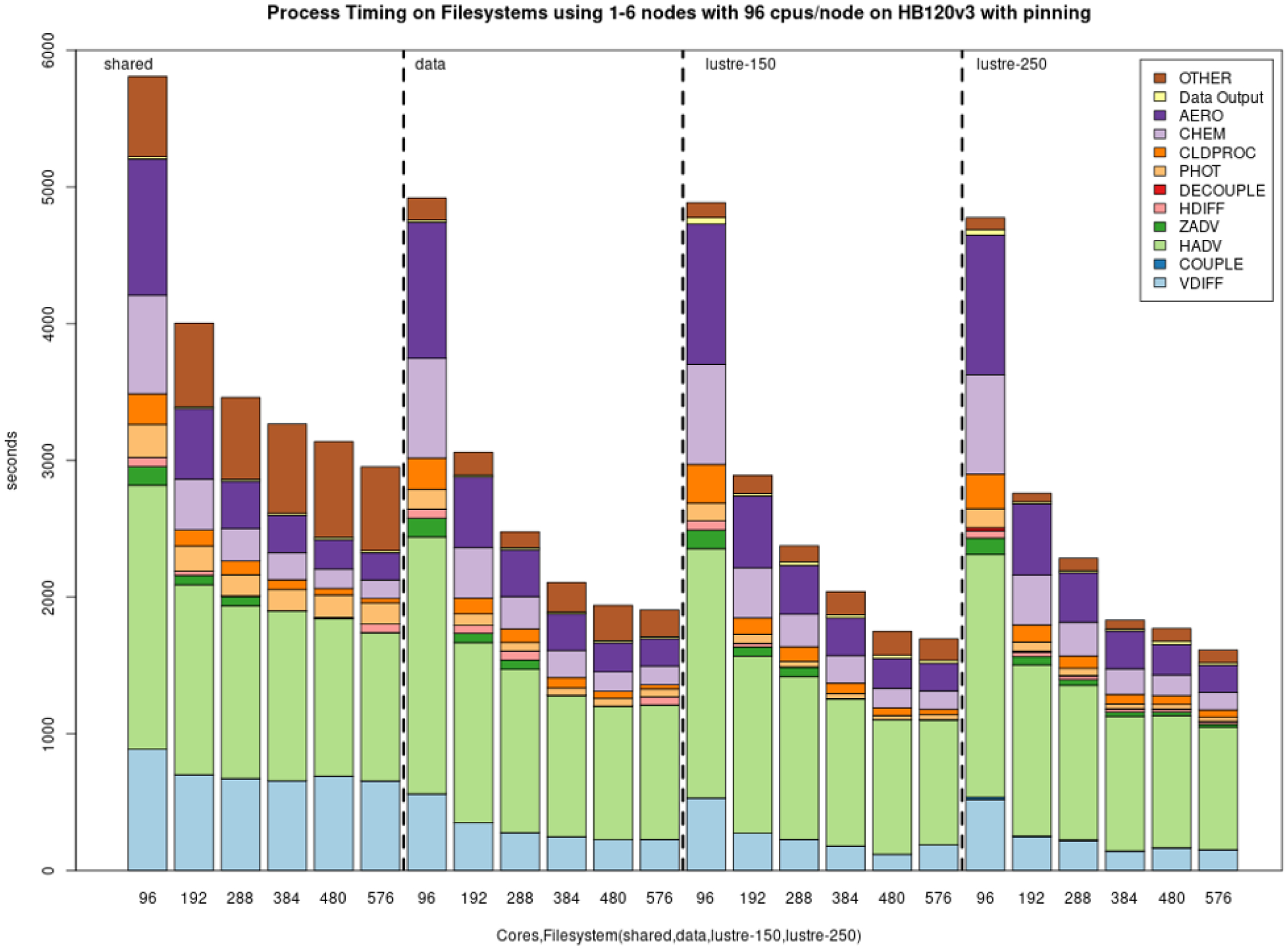
Performance of the cloud benchmark suite (CBS_limited) on Microsoft Azure CycleCloud environments using file systems (shared, data, Lustre 150 and Lustre 250) for I/O and code on /shared.

**Figure 11.**
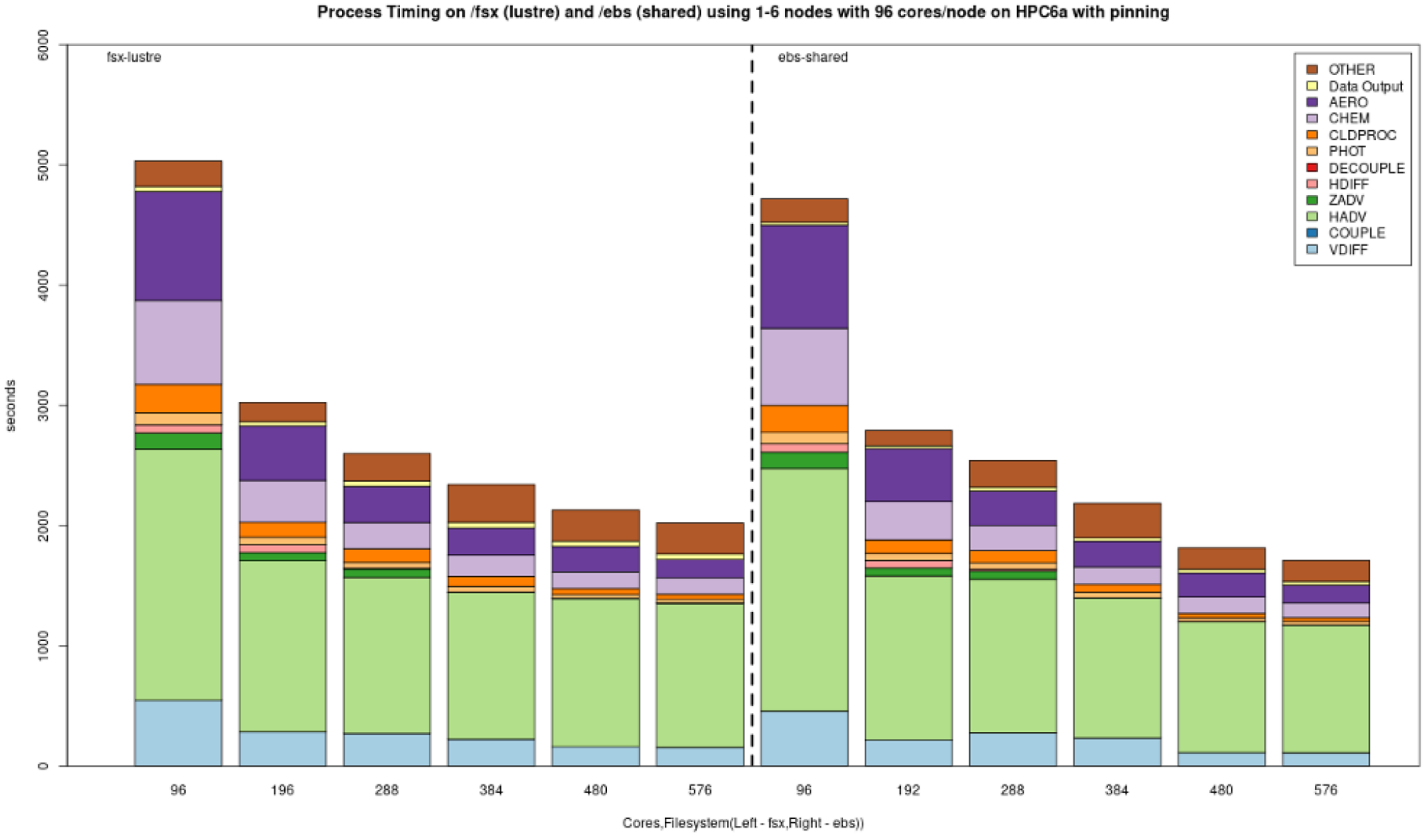
Performance of the cloud benchmark suite (CBS_limited) on AWS ParallelCluster environments using /fsx (lustre) and /ebs (shared) file system.

Figure 10 demonstrates the benchmark case results from simulations performed on Azure’s CycleCloud clusters employing one–six nodes and two different Lustre implementations, a faster (250 MB s^−1^ TiB^−1^) and a slower (150 MB s^−1^ TiB^−1^) tier, both of size 100 TiB and for the NFS share and the slower built-in NFS solution, respectively. Depending on the end-user cost and overall simulation needs, the slower solution can be a more cost-effective one, while the expensive option can be chosen when a faster turnaround time is necessary. In all cases, we observed diminishing performance gains when utilizing more than two nodes, with a plateau becoming apparent in the three–six-node region. Figure 11 provides the performance breakdown for AWS’s Lustre storage option and using the EBS (/shared) storage option. On AWS ParallelCluster, a scratch Lustre option (200 MB s^−1^ TiB^−1^) was used. Lustre appears to be comparable in both CSP implementations, with minor differences that can be attributed to the size of the file system that was provisioned (100 TiB on Azure CycleCloud, 1 TiB on AWS ParallelCluster) and the way the file system was parameterized (including stripe size) by different CSPs. EBS benchmarks were significantly faster, which makes it a potentially better alternative to Lustre for AWS instances. On AWS ParallelCluster, the Lustre file system is connected to the CMAS Center Open Data S3 bucket, and only the files that are used in the run script are copied from the S3 bucket to Lustre. This strategy is used to identify resources as non-blocking (non-critical) and load these only when needed, referred to as lazy loading. For the EBS benchmark, an AWS CLI script is used to copy the data from the S3 bucket to the EBS volume. The time taken to copy the data using the AWS CLI is higher (~ 15 min) than the time it takes for the data to be read from the S3 bucket by Lustre (~ 300 s), and these timings were not included since the data were preloaded to the file systems for these benchmarks.

#### 4.2.3 Results on multi-node clusters with different storage options saving 37 variables to the all-layer CONC file

**Figure 12.**
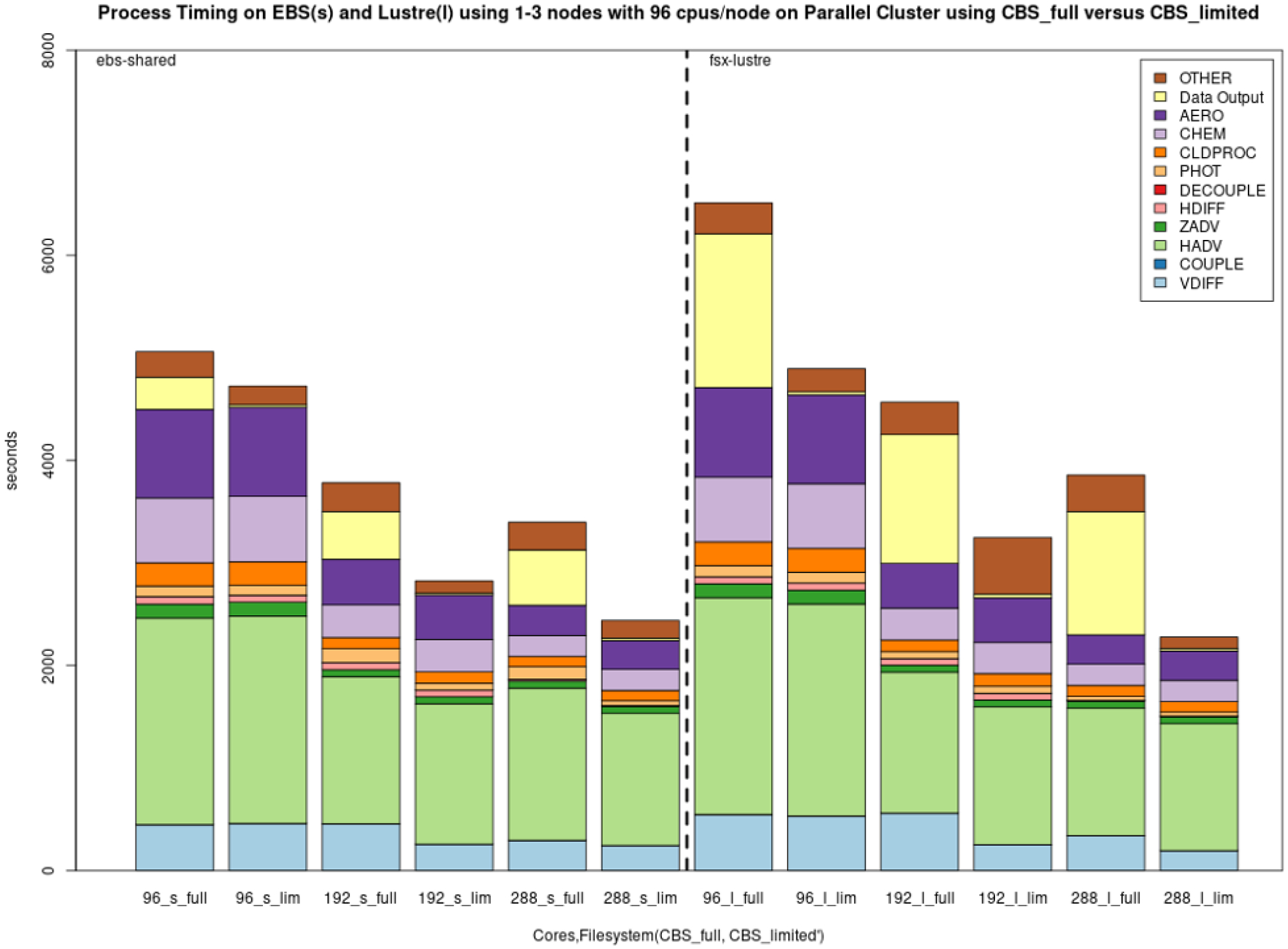
Performance of the 2 d cloud benchmark suite using 1–3 nodes with 96 CPUs/nodes on AWS ParallelCluster environments using full output (CBS_full) versus limited output (CBS_limited) on EBS (shared (s)) and Lustre (l).

Figure 12 shows the performance of the EBS (shared) and the Lustre file systems using 96, 192 and 288 cores on AWS when either a limited number of output variables and one layer or the full number of output variables and all layers are saved to the 3D CONC file (creating and saving the largest output file possible under the cloud benchmark case – CBS_full).

### 4.3 Cost analysis of compute nodes

Table 4 shows a comparison of the compute-only costs associated with an annual simulation based on the cloud benchmark suite with limited output file options. The setup was based on two-node cluster setups for both CSPs and the option of spot-pricing that was only available for Microsoft Azure. It should be noted that spot instances can be preempted, resulting in a termination risk that the user should be aware of when designing their implementation. The compute node hpc6a.48xlarge is not provided as a spot instance, as the on-demand price is significantly discounted (60 %). However, Amazon does offer spot prices for other compute nodes. This analysis used on-demand pricing options to uniformly evaluate both systems. Users will need to implement code to check-point and recover from a simulation termination if they choose to use spot-pricing or be willing to restart simulations if spot instances are terminated.

### 4.4 Results of using DESID module for emission-scaling sensitivity studies

**Figure 13.**
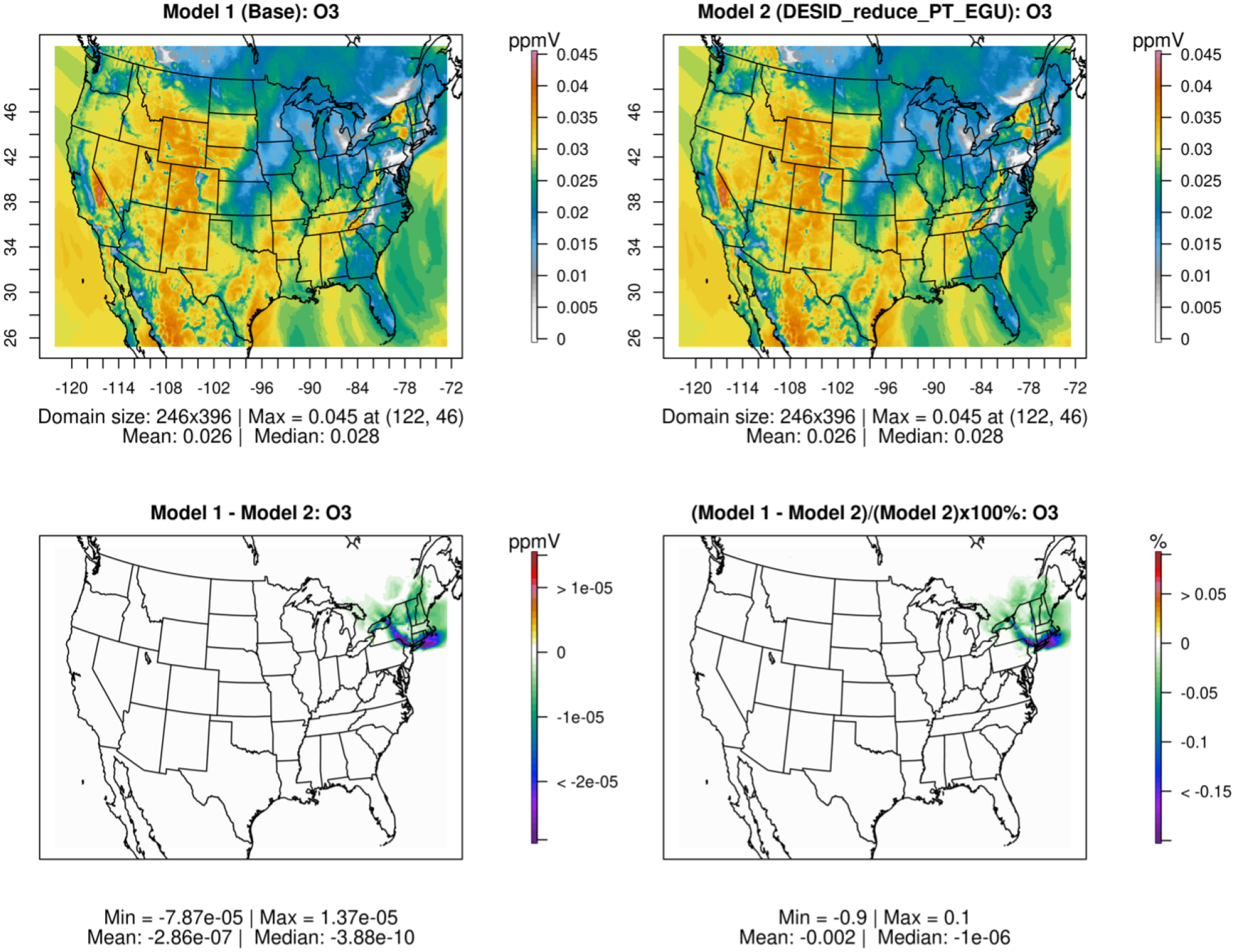
Model-to-model comparison for a base case of CMAQ version 5.3.3 and a sensitivity case where DESID was used to reduce the point-source electric generating unit (PT_EGU) emissions in New York State by 25 %.

Online tutorials are available for CMAQ version 5.4 with instructions for running baseline and sensitivity examples using DESID. Model-to-model comparison plots using spatial and time series analysis scripts in R and Python are also provided and demonstrated. Figure 13 shows the model-to-model mean spatial plots, absolute difference plot and percent difference plot for ozone from a base simulation and a sensitivity case where the DESID module was used to reduce the emissions from power plants in New York State (Foley, 2024).

## 5 Discussion

### 5.1 Benefits of proposed cloud-based implementation

Previous efforts of bringing CMAQ to the cloud demonstrated the potential of packaging the model along with other components as a standalone service optimized for small benchmark domains and low-cost VMs (Zhang et al., 2019). Currently, running complex, computationally demanding models on the cloud presents new options for optimizing workflow, performance and costs with access to HPC resources. A major implication is a fast deployment of such infrastructures with precompiled software snapshots and preloaded data that are easy to configure and customize according to the user needs. This work evaluates how to run CMAQ on two CSPs using their cluster management tools (ParallelCluster and CycleCloud) and illustrates several issues that should be considered for building HPC clusters on the cloud. We observed that, despite their differences, both AWS and Azure performed similarly and had comparable performance to on-site HPC implementations used in earlier phases of this work. The online tutorials provide guidance for selecting CSP cost-effective options (compute nodes, storage and high-performance networking) for the current CONUS benchmark suite and can be used as a guide for benchmarking more demanding CMAQ applications, such as the coupled model (WRF-CMAQ) (Wong et al., 2012), the Integrated Source Apportionment Method (CMAQ-ISAM) (Cohan and Napelenok, 2011), or simulations with higher horizontal or vertical grid resolution.

### 5.2 Impact of storage options and process pinning on CSPs

The choice of a storage option is shown to have an important impact on simulation run times. Figure 7 shows the best performance on NVMe drives, which are only available on single virtual machines. To fully utilize the potential of HPC solutions, the Lustre storage option is advisable for the Azure ecosystem. For AWS, EBS offers a cost-effective alternative to Lustre. However additional data transfers may be required (e.g., copying input and output data to/from the S3 bucket) in the workflow if the ParallelCluster is configured to have the EBS volumes deleted when the cluster is terminated. Scaling performance was improved when both the code and the data files reside on /lustre and may also improve if both the code and the I/O are on local storage (/nvme) or Azure file share (/data). The effect of process pinning resulted in modestly improved timings on both EBS and Lustre on AWS ParallelCluster. Process pinning resulted in significant timing improvements for Lustre on Azure CycleCloud. Process pinning resulted in improved performance on /shared, /data and /lustre using Azure’s CycleCloud, with the best performance being on Lustre.

### 5.3 Scalability

A key issue that is brought up in the Results section is the model scalability, which exhibits a diminishing return as the systems are scaled out across more cores and nodes. In general, scalability depends on the domain characteristics (domain size, resolution) and the hardware. Domain decomposition can significantly reduce performance when the domain is highly decomposed; i.e., only a few grid cells are assigned to each core. While our results focus on a fixed-size cloud benchmark suite, we expect improved scalability with a higher-resolution domain, as it would increase the workload per core. Compared to typical WRF benchmarks where a 2.5 km × 2.5 km resolution CONUS domain (63 million grid cells) is considered a typical case that scales well up to a few hundred cores, the 12 km × 12 km CMAQ CBS (97 000 grid cells) is comparatively too small of a problem to scale similarly.

### 5.4 Future research recommendations

The online tutorials and documentation include recommendations for future work such as (1) running the benchmark using new releases of virtual machines (Elastic Compute Cloud (EC2) instances or Microsoft Azure VMs), (2) building with the EPYC processor including Standard_HB176rs_v4 on Azure CycleCloud and on new releases of the Arm-based AWS Graviton 3 processor using c7g.16xlarge on ParallelCluster, and (3) running the benchmark using a parallel I/O API implementation and other efficiency improvements in the source code that can be specific to the compiler and processor architecture. The impact on performance needs to be examined after each model release and for each model configuration and input platform data, which vary by year and model parameterization.

### 5.5 Using a cloud service provider versus on-premise compute servers

The choice of conducting simulations on CSPs versus an on-premise option comes down to the cost and resources of the on-premise option and the specifics of the work to be accomplished. In some cases, organizational investments to support computational and data needs may effectively subsidize the cost of an on-premise solution. Even when an on-premise option is available, it may be reasonable to utilize both on-premise and CSP services to take advantage of the flexibility and scalability of building custom infrastructure and workflows within a CSP. Our cost estimates for the cloud benchmark suite support the conclusion by Zhuang et al. (2020) that atmospheric modeling in the cloud can be a cost-competitive alternative to more traditional HPC systems.

## 6 Conclusions

This work provides reproducible workflows to facilitate provisioning of HPC clusters on the cloud, setting up and running CMAQ, and using performance analysis tools and profilers to optimize the HPC systems available from CSPs. The very nature of cloud implementations comes with the advantage that CSPs are continuously offering advancements in compute, memory and storage resources. Different CSPs use different hardware versions (EPYC processor versions), with a different hypervisor on different Nitro chip and SLURM implementations, which result in a different number of cores available per node. Azure provided manual pinning instructions that required a detailed understanding of the CPU architecture and SLURM scheduler. In the case of AWS, the implementation resulted in fewer cores/nodes available to the user (96 versus 128) but automatically bound the specific process to a core and did not require additional fine-tuning steps (manual pinning had less impact on timings). CMAQ is also continuously under development, and while the method presented here used CMAQ version 5.3.3 with the 12US2 benchmark and the CB6 mechanism, it may be extended to compile and run new versions of CMAQ, new mechanisms such as the Community Regional Atmospheric Chemistry Multiphase Mechanism (CRACMM) (Pye et al., 2023), and other configurations including WRF-CMAQ and CMAQ-ISAM. Our HPC in the cloud paradigm allows researchers to improve their workflow and access a menu of specialized HPC resources offered by cloud computing vendors resulting in a faster time to the solution. These tutorials by the CMAS community are designed to facilitate the use of best practices for cloud HPC provisioning, increase crossinstitution collaborations, and improve efficiency in code development and deployment cycles.

## Figures and Tables

**Table 1.** CMAQ configuration and storage needs for the CONUS case benchmark suite.

CMAQ version	5.3.3 with code modifications to fix cloud-specific bugs
OS	Linux, processor 64-bit ×86 (Ubuntu on AWS, AlmaLinux on Azure)
Memory	> 1 GB RAM per CPU core
Storage	Disk space requirement for the 2 d benchmark suite is 250 GB: 44 GB input data and 170 GB output data (output files included concentrations for all species, all layers) (CBS_full) or 18 GB output data (for CONC file limited to 12 species, 1 layer) (CBS_limited)
Domain (no. columns × no. rows × no. layers)	396 × 246 × 35
Horizontal domain resolution	12km × 12km
Temporal resolution of output	Hourly
Temporal duration	2 d
Chemical mechanism	cb6r3_ae7_aq

**Table 2.** Overview of system configurations and technical capabilities for the two HPC systems that were used for benchmarking.

HPC test system description		
Cloud service provider	Microsoft Azure	Amazon Web Services
Service name	CycleCloud	ParallelCluster
VM name	Standard_HB120rs_v3	hpc6a.48xlarge
Processor	AMD EPYC 7V73X	AMD EPYC 7R13
CPU cores available	120	96
CPU speed (MHz)	1846	2650
Memory (GiB)	461	384
L3 cache memory (MB)	96	192
Network bandwidth (Gb s^−1^)	200 (NVIDIA HDR InfiniBand)	100 (Elastic Fabric Adapter – EFA)

**Table 3.** Overview of storage options for the two HPC systems that were used for benchmarking.

Storage options		
Cloud service provider	Microsoft Azure	Amazon Web Services
Service name	CycleCloud	ParallelCluster
Storage option 1 (/local)	Local NVMe SSDs in RAID 0 (2 · 960 GB NVMe – block)	NA
Storage option 2 (/shared)	Built-in NFS: P30 tier; provisioned IOPS: 5000; provisioned throughput: 200 MB s^−1^ TiB^−1^	Elastic Block Storage (EBS) – general purpose volumes (gp3); provisioned IOPS: 3000; provisioned throughput: 1000 MiBs^−1^
Storage option 3 (/data)	NFS file share: max IOPS 4024; burst IOPS: 10 000; throughput rate: 203 MB s^−1^	NA
Storage option 4 (/lustre)	Lustre 150 – size 128 TB: performance profile – 150 MB s^−1^ TiB^−1^; Lustre 250 – size 128 TB: performance profile – 250 MB s^−1^ TiB^−1^	Lustre SCRATCH_2 option: size – 1 TB; network throughput: 200 (1300 burst) MBs^−1^ TiB^−1^; 240 MBs^−1^; disk throughput: 200 MB s^−1^ TiB^−1^ (read), 100MB s^−1^ TiB^−1^ (write)

NA: not available

**Table 4.** Comparison of the compute costs for performing an annual simulation based on the cloud benchmark suite (CBS_limited) on two-node clusters with on-demand and spot-pricing tiers. Note that these costs are indicative and do not include any other components of the cluster (storage, head node, etc.).

Compute node	Cores	Nodes	Pricing	Cost per node (USD)	CBS wall time (hour)	Extrapolated annual cost	Days to complete annual simulation of CBS
HB120v3	192	2	On-demand	3.6 h^−1^	0.767	USD1007	5.83
HB120v3	192	2	Spot	1.4 h^−1^	0.767	USD392	5.83
hpc6a.48xl	192	2	On-demand	2.88 h^−1^	0.839	USD883	6.4

**Figure A1.**
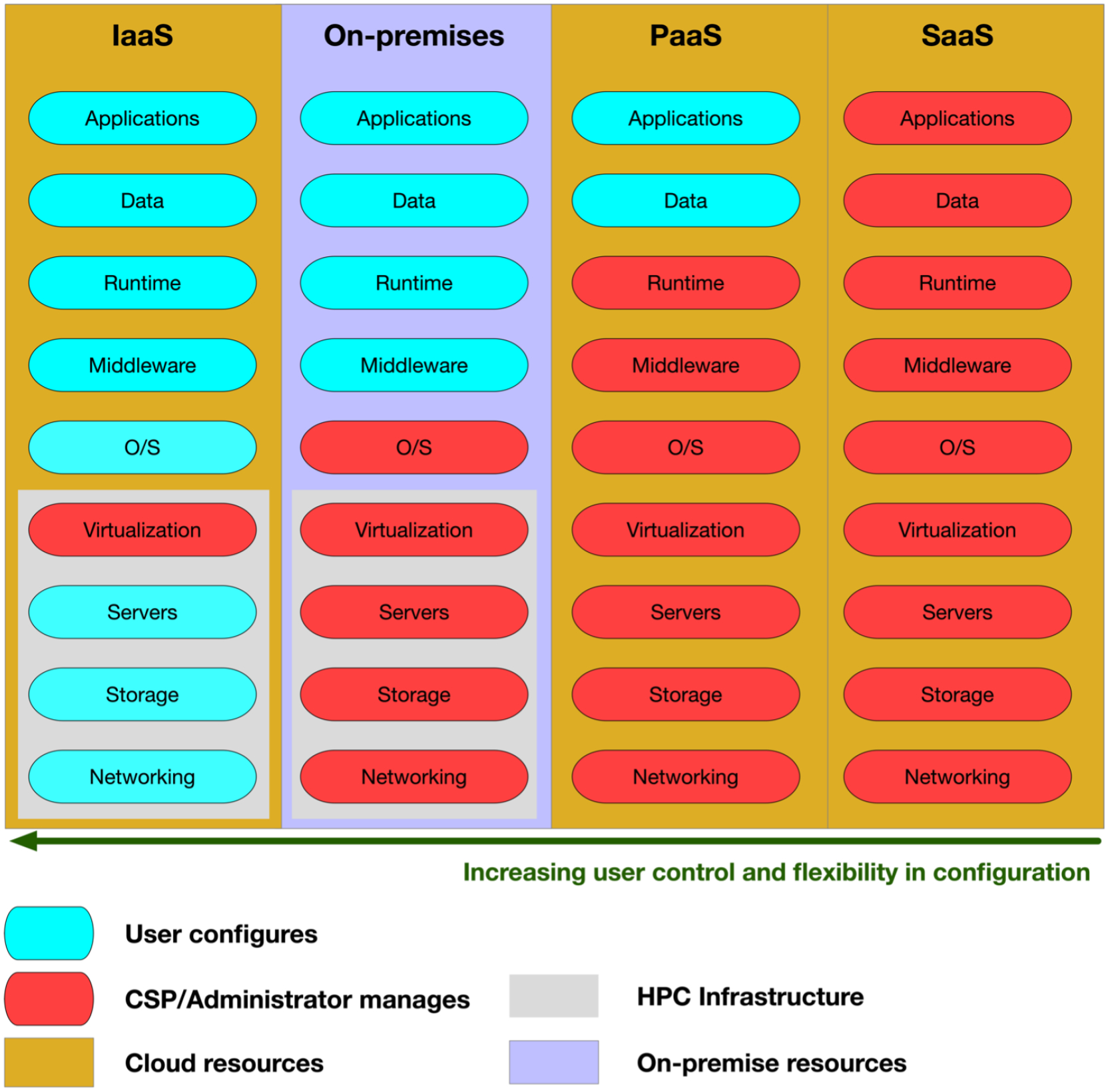
Architecture of on-site and cloud-based services demonstrating the degree of end-user control.

**Figure B1.**
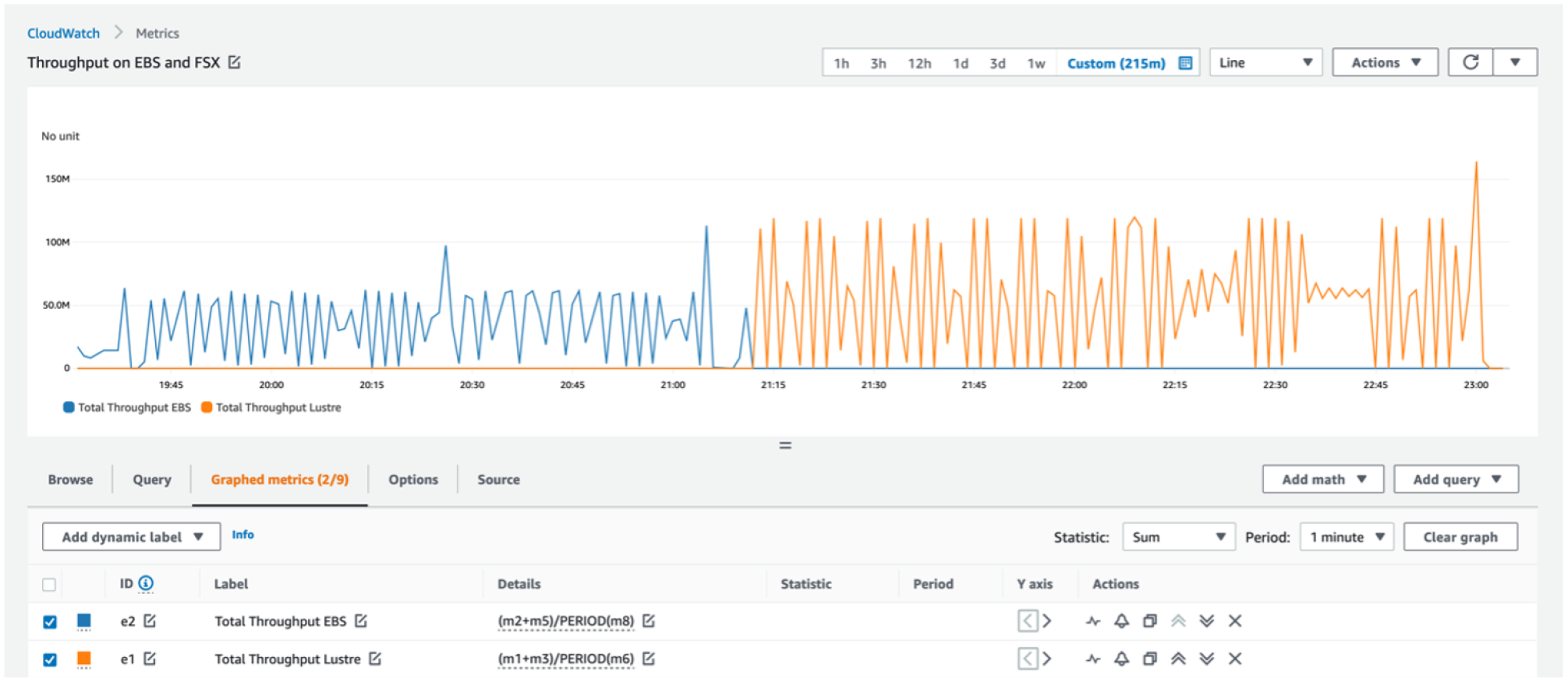
Throughput measurement of the first day of the cloud benchmark suite using 96 cores on ParallelCluster using different file systems – EBS (blue) and Lustre (orange) – for full output (all variables, all layers) in the CONC file (CBS_full). Credit: Amazon^®^ CloudWatch.

**Figure B2.**
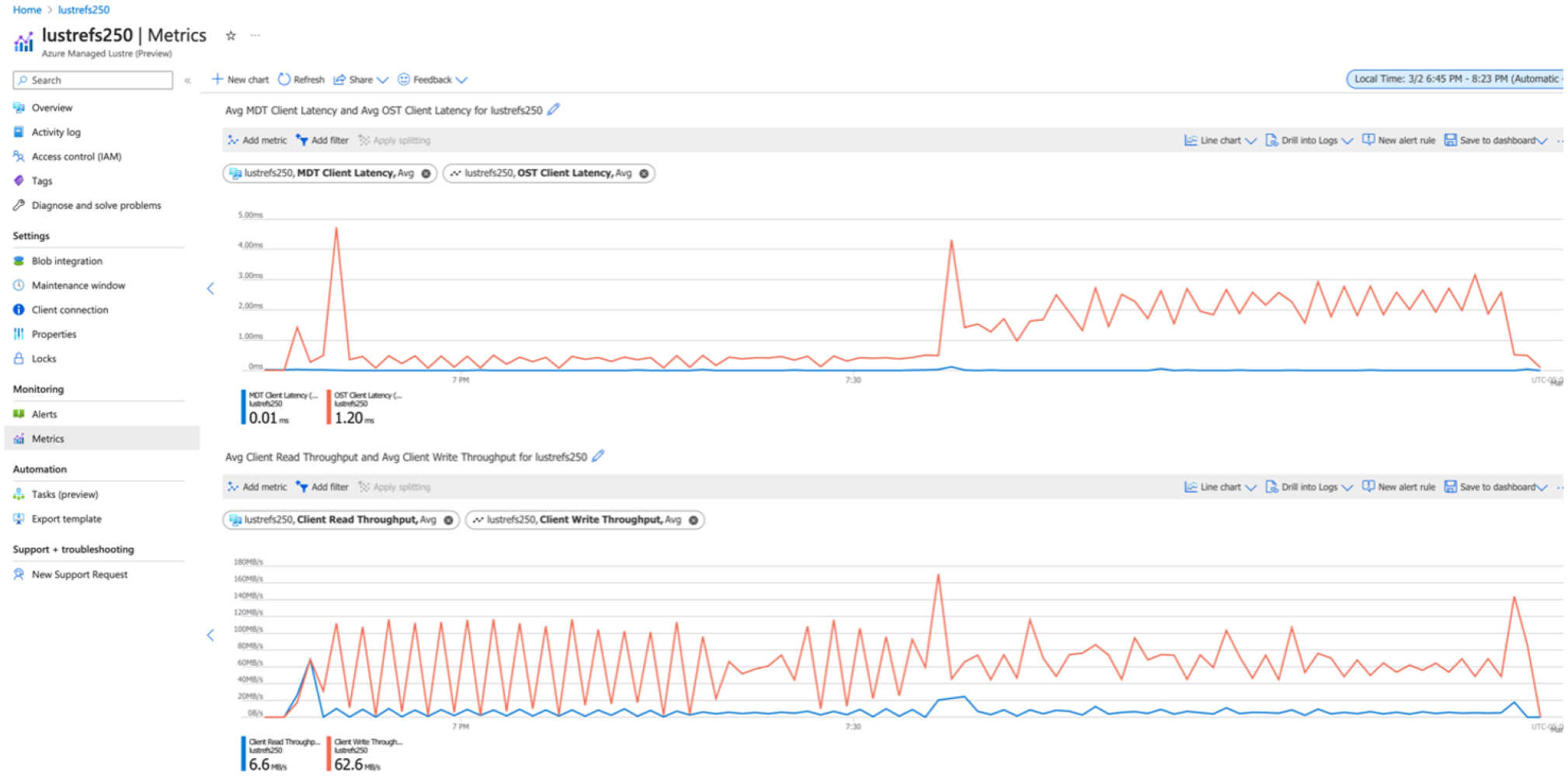
Cloud metric latency and throughput measurement of data read and data write for the cloud benchmark suite using 96 cores on CycleCloud using the Lustre file system for full output (all variables, all layers) in the CONC file for the cloud benchmark suite (CBS_full). Credit: Azure^®^ Monitor.

**Figure B3.**
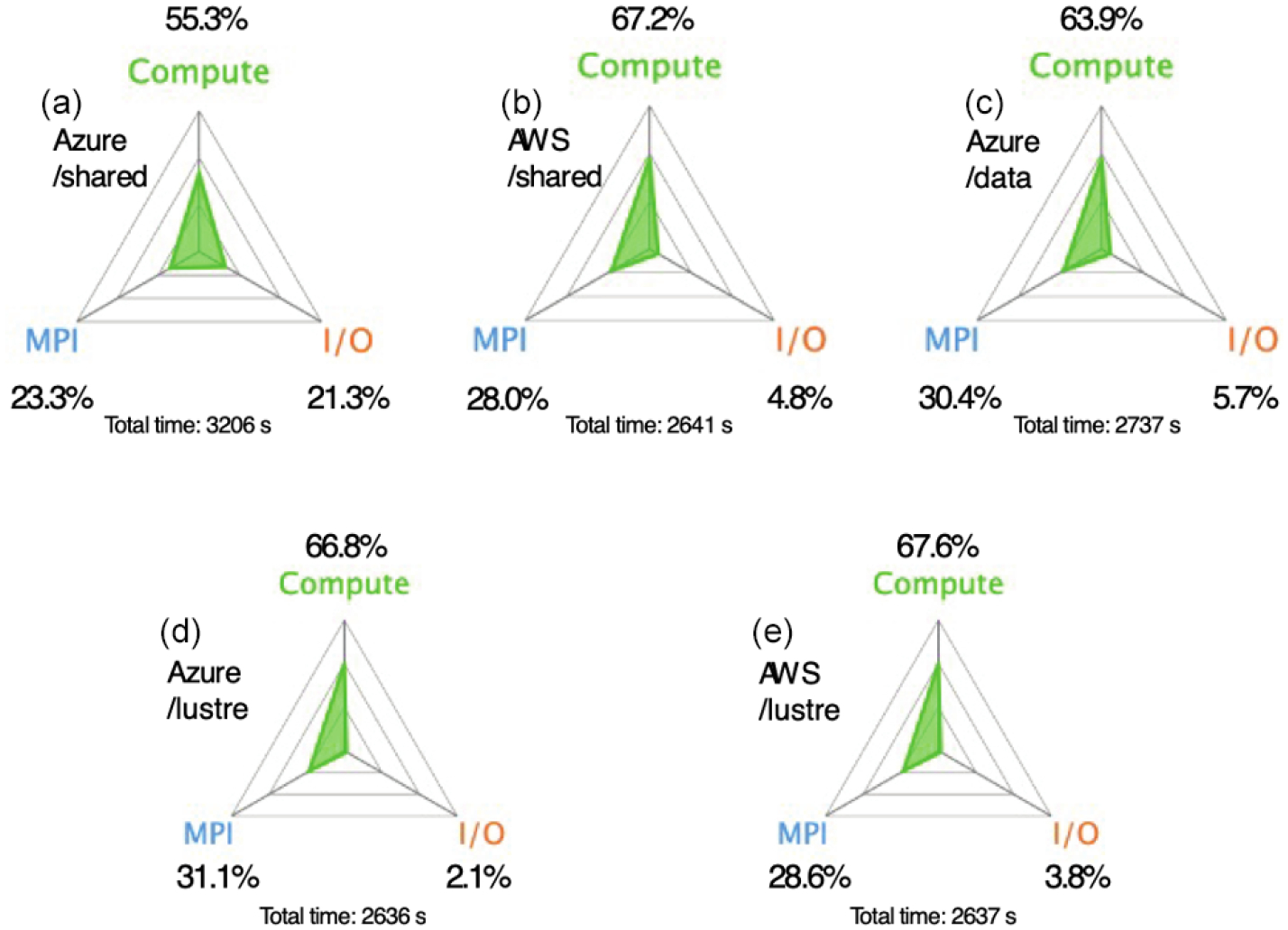
Code profiler performance analysis for the first day of the cloud benchmark suite (CBS_limited) on AWS’s ParallelCluster and Azure’s CycleCloud using different storage options (a Azure /shared, b AWS /shared, c Azure /data, d Azure /lustre and e AWS /lustre). Credit: ARM^®^ MAP.

**Figure B4.**
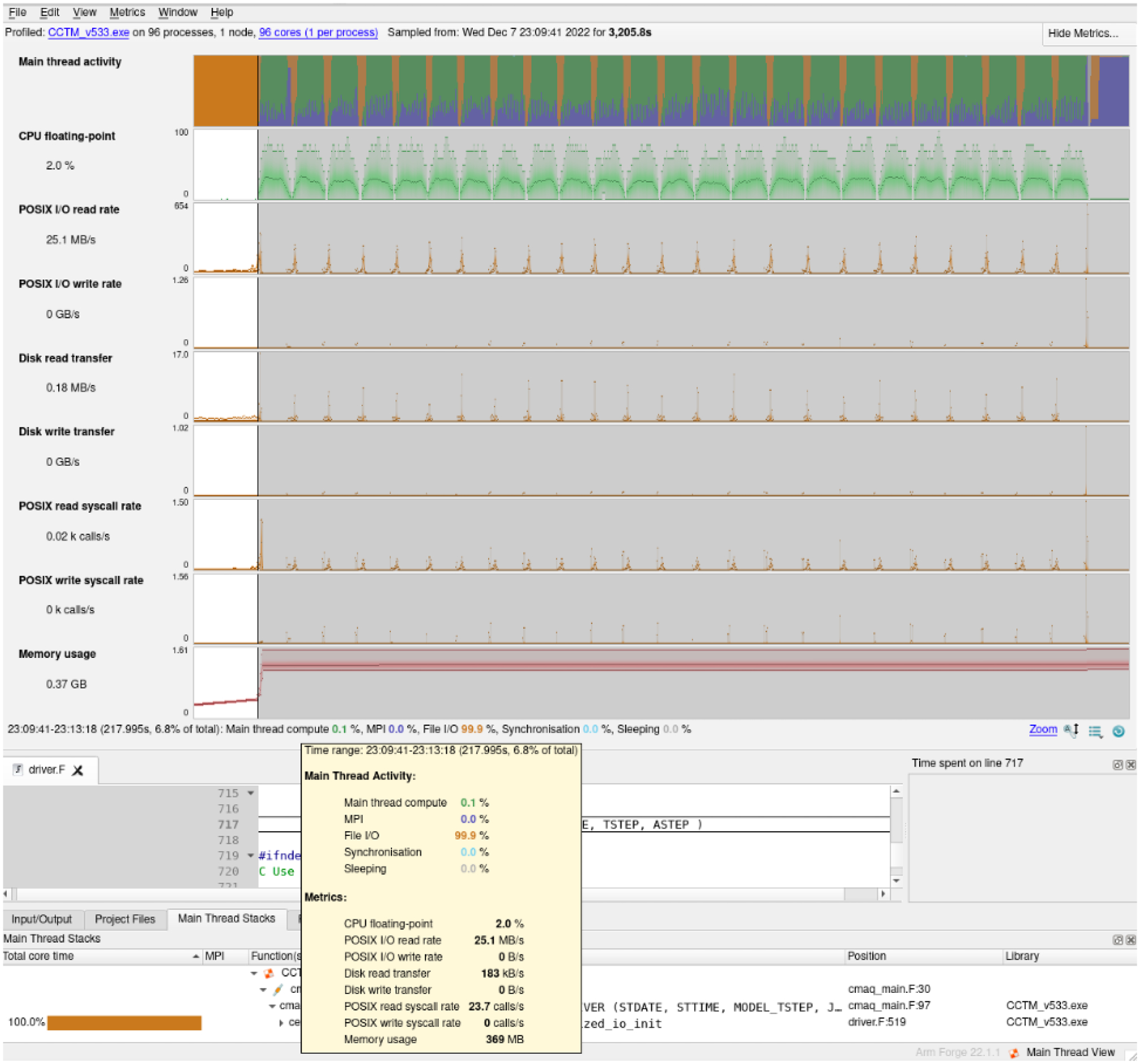
Code profiler single-day performance analysis for Azure’s CycleCloud using the NFS /shared file system. Credit: ARM^®^ MAP.

**Figure B5.**
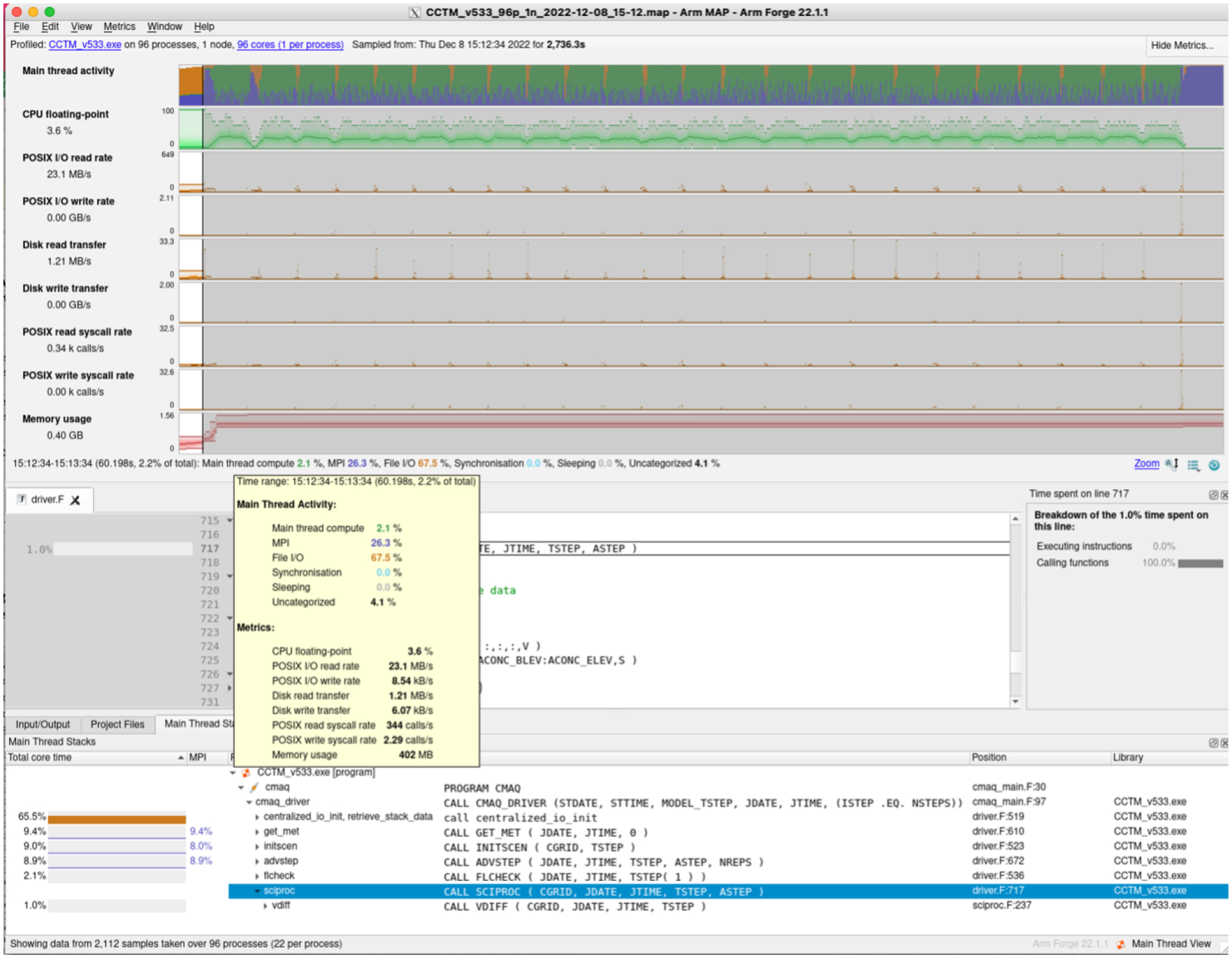
Code profiler single-day performance analysis for Azure’s CycleCloud using the /data file system. Credit: ARM^®^ MAP.

**Figure B6.**
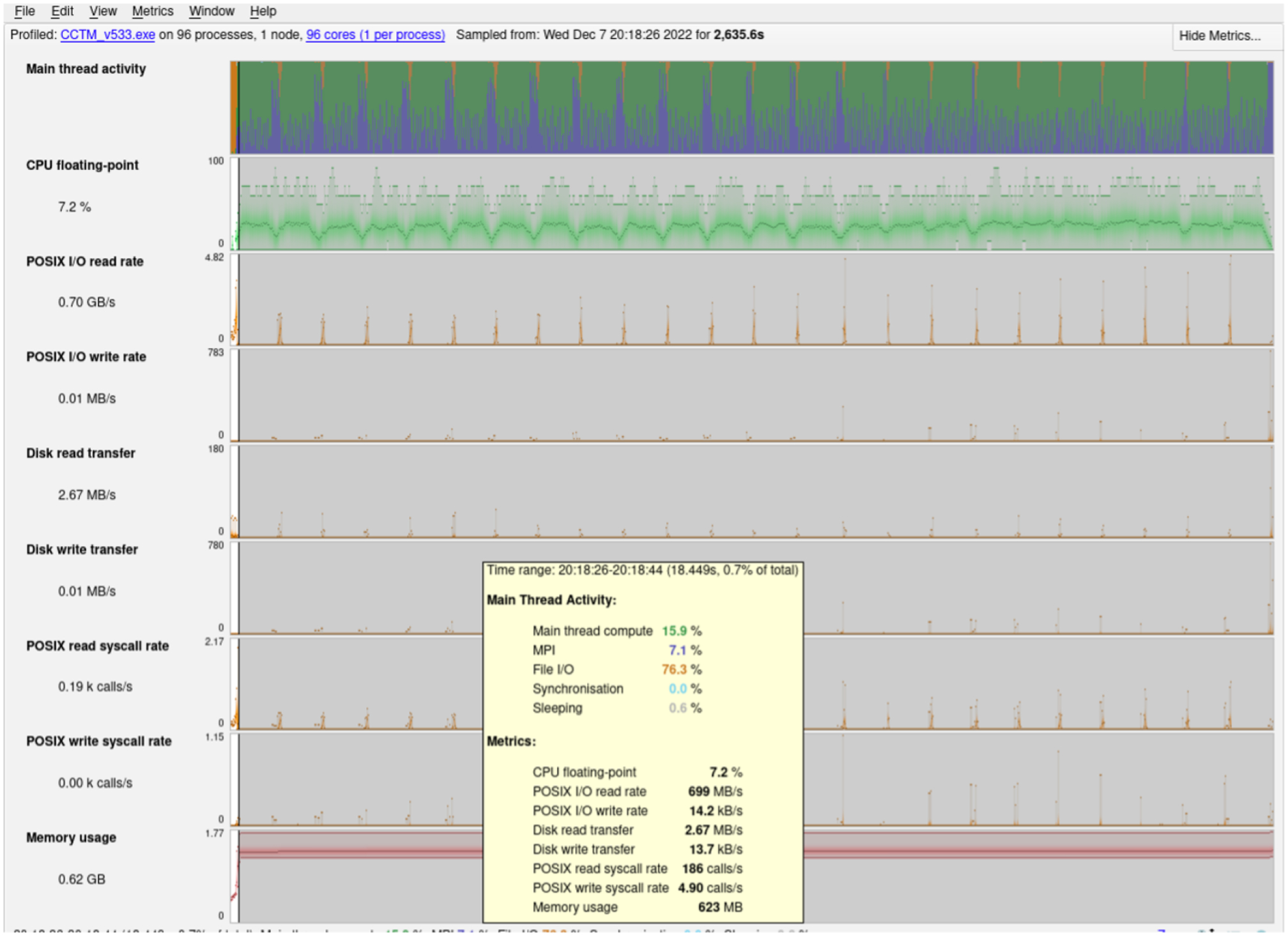
Code profiler single-day performance analysis for Azure’s CycleCloud using the Azure-managed Lustre file system. Credit: ARM^®^ MAP.

## Data Availability

The code for CMAQ is available and referenced at https://github.com/USEPA/CMAQ/ (last access: 20 June 2024) and https://doi.org/10.5281/zenodo.5213949 (US EPA Office of Research and Development, 2021). The code for the AWS cloud implementation is available and referenced at https://github.com/CMASCenter/pcluster-cmaq/tree/CMAQv5.3.3 (last access: 20 June 2024) and https://doi.org/10.5281/zenodo.10696908 (Adams et al., 2024a). The code for the Azure cloud implementation is available and referenced at https://github.com/CMASCenter/cyclecloud-cmaq/tree/CMAQv5.3.3 (last access: 20 June 2024) and https://doi.org/10.5281/zenodo.10696804 (Adams and Efstathiou, 2024a). Data inputs for the benchmark suite are available and referenced at https://registry.opendata.aws/cmas-data-warehouse/ (last access: 20 June 2024) and https://doi.org/10.15139/S3/CFU9UL (Adams, 2024). Tutorials with instructions on running CMAQ version 5.3.3 and above on the cloud are available through https://cyclecloud-cmaq.readthedocs.io/en/cmaqv5.3.3/ (Adams and Efstathiou, 2024b) and https://pcluster-cmaq.readthedocs.io/en/cmaqv5.3.3/ (Adams et al., 2024b).

## Appendix A: HPC architectures and CSP services

A growing number of companies ranging from large enterprises such as Amazon, Microsoft and Google to a spectrum of cloud-focused computer firms have a strong presence with evolving portfolios in what is broadly defined as “public cloud infrastructure”. Typically, cloud computing is provided through at least four types of services summarized in Fig. A1: infrastructure as a service (IaaS), platform as a service (PaaS), software as a service (SaaS) and data as a service (DaaS) (Mell and Grance, 2011; Chang et al., 2010; Yuan, 2016). IaaS products (Amazon Web Services, Microsoft Azure, Google Cloud, etc.) allow organizations and end users to manage their system resources (i.e., servers, network, data storage) on the cloud. PaaS products (Windows Azure, Google app engine, etc.) allow businesses and developers to host, build and deploy consumer-facing apps. The most important contrast between IaaS and PaaS is that IaaS gives users more administrative control over operating systems and system resources, while PaaS gives consumers the ease of use of provided applications but limits access to choices about the operating system and system resources. SaaS products are among the most popular cloud computing services (Microsoft 365, Google Docs, Adobe cloud, etc.) offering out-of-the-box, simple solutions that usually target common end users and operate under a subscription model. DaaS, the least well-defined type of service, describes cloud-based software tools used for working with data, such as managing data in a data warehouse entity and processing or analyzing with software tools, typically enabled by SaaS technologies under a subscription model. HPC applications require administrative access to networking, hardware and storage configurations and therefore need infrastructure as a service (IaaS) and infrastructure as code (IaaC) levels of control that are provided by the ParallelCluster and CycleCloud cluster management services.

## Appendix B: Tools for storage performance monitoring

### B1 Amazon CloudWatch

Amazon CloudWatch (Amazon CloudWatch, 2024) and Azure Monitor (Azure Monitor, 2024) allow you to monitor the I/O throughput of a file system while running an application in real time. Amazon CloudWatch output shown in Fig. B1 can be used to further investigate the impact of storage on performance seen when writing additional model outputs (e.g., full layered 3D instantaneous model concentrations) to different storage options. While AWS CloudWatch shows higher throughput on Lustre than shared, the benchmark performance is faster on shared than Lustre. This may be due to larger disk caches or faster latencies on the EBS volume. The Lustre performance may be improved by using a persistent volume versus scratch volume that was used in this study.

### B2 Azure Monitor

Figure B2 displays the read and write throughput and client latency metrics from the Azure Monitor for the cloud benchmark suite using the Azure^®^-managed Lustre system (250 MB s^−1^). Insights into the I/O performance are facilitated by the web tools available from each CSP.

### B3 ARM^®^ MAP code profiler analysis for Azure CycleCloud and AWS ParallelCluster

As mentioned before, examining the main log and associated timing plots does not precisely capture the time spent in I/O tasks. The use of a code profiler such as the ARM^®^ MAP Profiler (ARM Ltd., 2022) can provide better insights into the model components, along with more detailed capture of the I/O tasks by different code routines. Results from applying the ARM^®^ MAP code profiler on a single-day benchmark simulation using the limited I/O benchmark for each CSP cluster and storage offering are presented in Fig. B3. We can clearly see a very similar behavior in both systems with the model using a Lustre file system. For /shared, AWS’s EBS solution is performing much better, with less time spent in I/O, which allows speeds closer to physical, “bare-metal” server equivalents. For Azure, the /shared volume has proportionately higher time spent in I/O and lower percentage spent on computation. In earlier tests without a profiler, the Azure NetApp Files (ANF) solution provided better I/O performance, but due to considerably higher cost due to a minimum file size of 4TB, we chose not to include an ANF setup in the profiler tests. The performance improvements going from /shared to /data and Lustre on Azure’s CycleCloud are also demonstrated by comparing Figs. B4, B5 and B6, where orange at the beginning of the run indicates the I/O portions that get reduced as we utilize faster storage options.
